# Functional expression of Δ12 fatty acid desaturase modulates thermoregulatory behaviour in *Drosophila*

**DOI:** 10.1038/s41598-020-68601-2

**Published:** 2020-07-16

**Authors:** Takuto Suito, Kohjiro Nagao, Kenichi Takeuchi, Naoto Juni, Yuji Hara, Masato Umeda

**Affiliations:** 1 Department of Synthetic Chemistry and Biological Chemistry, Graduate School of Engineering, Kyoto University, A4-212 Katsura, Nishikyo-ku, Kyoto, 615-8510 Japan; 2 Present Address: Division of Thermal Biology, Exploratory Research Center On Life and Living Systems (ExCELLS), National Institutes of Natural Sciences, 5-1 Higashiyama, Myodaiji-cho, Okazaki, Aichi 444-8787 Japan

**Keywords:** Biochemistry, Lipids, Fatty acids, Phospholipids

## Abstract

Polyunsaturated fatty acids (PUFAs) play crucial roles in adaptation to cold environments in a wide variety of animals and plants. However, the mechanisms by which PUFAs affect thermoregulatory behaviour remain elusive. Thus, we investigated the roles of PUFAs in thermoregulatory behaviour of *Drosophila melanogaster*. To this end, we generated transgenic flies expressing *Caenorhabditis elegans* Δ12 fatty acid desaturase (FAT-2), which converts mono-unsaturated fatty acids to PUFAs such as linoleic acid [C18:2 (*n*-6)] and linolenic acid [C18:3 (*n*-3)]. Neuron-specific expression of FAT-2 using the GAL4/UAS expression system led to increased contents of C18:2 (*n*-6)-containing phospholipids in central nerve system (CNS) and caused significant decreases in preferred temperature of third instar larvae. In genetic screening and calcium imaging analyses of thermoreceptor-expressing neurons, we demonstrated that ectopic expression of FAT-2 in TRPA1-expressing neurons led to decreases in preferred temperature by modulating neuronal activity. We conclude that functional expression of FAT-2 in a subset of neurons changes the thermoregulatory behaviour of *D. melanogaster*, likely by modulating quantities of PUFA-containing phospholipids in neuronal cell membranes.

## Introduction

Polyunsaturated fatty acids (PUFAs) contain multiple double bonds in their hydrocarbon chains and, as bioactive lipids, regulate various animal physiological functions, such as those relating to immunity, reproduction and energy metabolism^1,2^. PUFAs have also been implicated in thermal adaptation, particularly cold adaptation, because the *cis* double bonds in PUFAs create the “kinks” in hydrocarbon chain structures that reduce the packing of phospholipids, thus increasing membrane fluidity at low temperatures^3,4^. For instance, ectothermic fish that live in low temperatures have high PUFA contents in phospholipids^5,6^, facilitating membrane fluidity^7^ and modulating the activities of membrane proteins, such as Na^+^–K^+^ ATPase^8^, in cold environments.

Most organisms produce mono-unsaturated fatty acids from saturated fatty acids through the actions of Δ9 fatty acid desaturases^9–13^. In mammals, PUFAs such as arachidonic acid [C20:4 (*n*-6)] and eicosapentaenoic acid [C20:5 (*n*-3)] are produced from linoleic acid [C18:2 (*n*-6)] and α-linolenic acid [C18:3 (*n*-3)], respectively, by Δ5 and Δ6 fatty acid desaturases. In contrast, Δ12 fatty acid desaturases that convert oleic acid [C18:1 (*n*-9)] to C18:2 (*n*-6), such as those in nematode *Caenorhabditis elegans*^14^, American cockroach^15^ and cricket^16^, are found in few animals. Therefore, most animals have to obtain PUFAs, such as C18:2 (*n*-6) and C18:3 (*n*-3), from their diet. In bacteria^17^, protozoa^18^, algae^19,20^ and plants^21,22^, Δ12 fatty acid desaturase can be reportedly induced following exposures to low temperatures. Hence, its biosynthetic product C18:2 (*n*-6) is thought to play crucial roles in low-temperature acclimation. *C. elegans* synthesises PUFAs using seven desaturases (FAT-1, FAT-2, FAT-3, FAT-4, FAT-5, FAT-6, FAT-7)^23,24^, and the *fat-2* gene encodes the sole Δ12 fatty acid desaturase. FAT-2 has a bifunctional ∆12/∆15-desaturase activity that produces both C18:2 (*n*-6) and C18:3 (*n*-3) from C18:1 (*n-*9)^25^. The enzyme also has broad substrate specificity with respect to fatty acyl chain lengths, which can range from C14 to C18^25^. Accordingly, defective expression of *fat-2* resulted in significant reductions in PUFA contents and caused various defects such as slow growth, abnormal body shape, sluggish movement, cuticle defects and reduced brood size^14^.

*Drosophila melanogaster* has been used as a model to evaluate the role of PUFAs in cold acclimation. As shown in other species, acclimation to low temperatures increased the proportions of PUFA-containing phospholipids in the cell membranes^26–29^, and these have been shown to contribute to the modulation of membrane fluidity and improve their development and survival in cold environments^30^. Recently, it was reported that breeding of flies at 12 °C switches dietary preference from yeast to PUFA-containing plant foods^30^. Because feeding behaviour is altered to modulate PUFA contents in response to temperature changes, we hypothesised that other temperature-related behaviour is affected by PUFA contents.

Thermoregulation is accomplished by temperature-sensing followed by effector reactions^31,32^. Behavioral thermoregulation (migrating to environments with more comfortable temperatures) is an essential part of the effector reactions in *D. melanogaster*^33^, because the body temperature of ectotherms is strongly affected by environmental temperature. Therefore, we focused on the behavioral thermoregulation in *Drosophila*. Previously, we reported that the Dystroglycan mutant fly *atsugari* showed cold-seeking behaviour with enhanced mitochondrial oxidative energy metabolism^34^; however, the factors that influence behavioral thermoregulation are not completely understood.

In this study, we investigated the roles of PUFAs in thermoregulatory behaviour and characterised the underlying molecular mechanisms. Although PUFAs have been shown to play important roles in the visual system^35^, synaptic functions^36^ and follicle maturation^37^ in *D. melanogaster*, the role of PUFAs in thermoregulatory behaviour has not been reported. Because *D. melanogaster* cannot synthesise PUFAs de novo, we established a transgenic *D. melanogaster* strain that expresses *C. elegans* FAT-2 under the control of the galactose-responsive transcription factor (GAL4)/upstream activating sequence (UAS) system. Then, we examined the effects of tissue- and cell type-specific expression of FAT-2 on thermoregulatory behaviour. From these data, we describe mechanisms how PUFAs affect thermoregulatory behaviour in *D. melanogaster*.

## Results

### Cold acclimation alters fatty acid compositions and thermoregulatory behaviour in *D. melanogaster*

**Figure 1:**
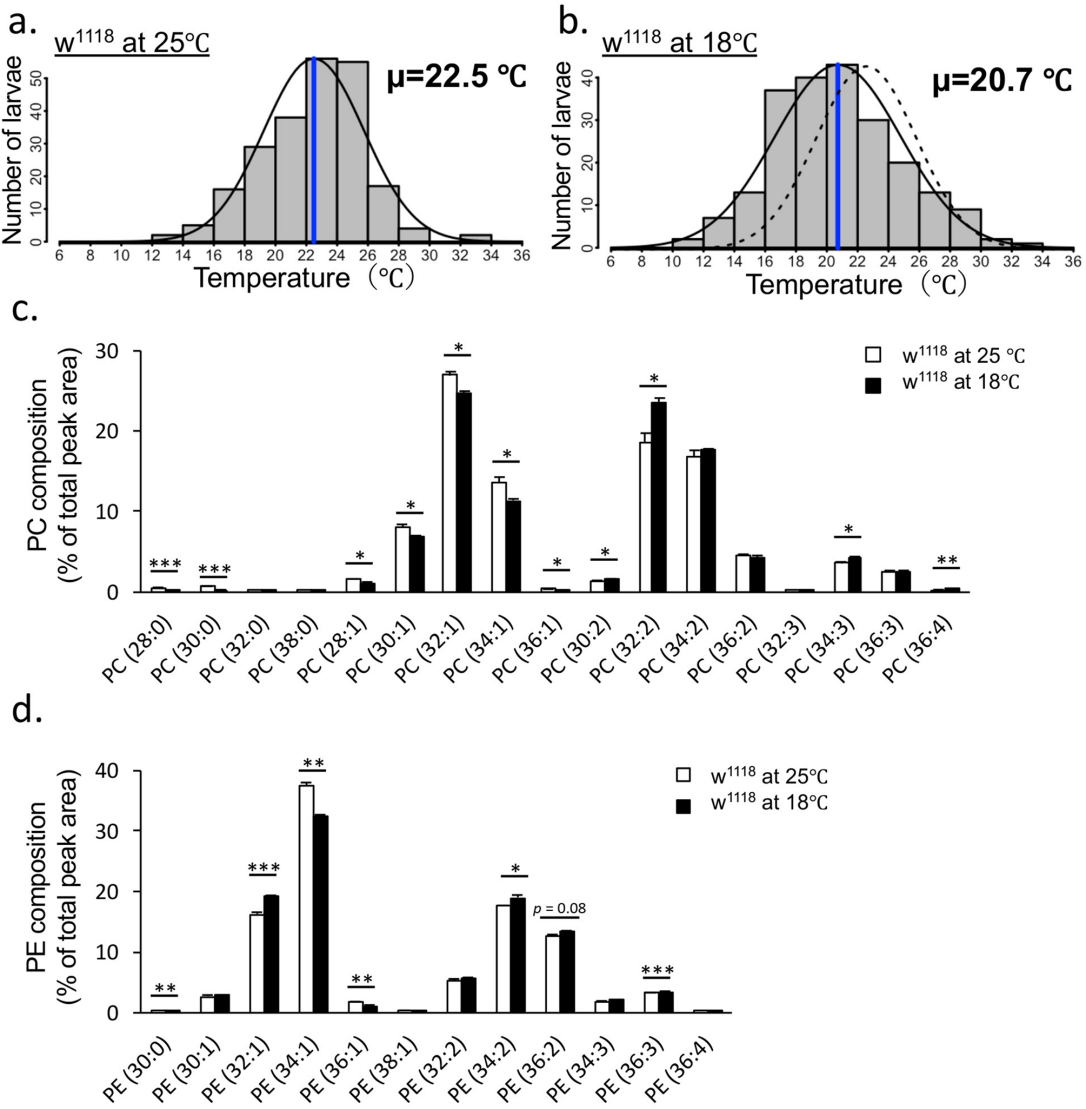
Cold exposure-induced changes in temperature preference and lipid compositions of third instar *D. melanogaster* larvae. Comparison of temperature preference of the w^1118^ wandering third instar larvae continuously cultured at 25 °C (n = 6) (**a**) and exposed at 18 °C for 1 day (n = 6) (**b**). The histogram shows distributions of the third instar larvae on the thermal gradient plate. The distribution curve is denoted by a black solid line and the average temperature preference is shown as a blue vertical line. The dotted curve represents the distribution of w^1118^ control larvae cultured at 25 °C. The numerical analyses of data are also shown in Supplementary Table S1. The proportions of phosphatidylcholine (PC) (**c**) and phosphatidylethanolamine (PE) (**d**) molecules in w^1118^ larvae continuously cultured at 25 °C (white bar, n = 3) and w^1118^ larvae exposed to 18 °C for 1 day (black bar, n = 3) were analysed using LC–MS/MS. Phospholipid molecules are shown in the format PC (X:Y) or PE (X:Y), where X denotes the total number of acyl chain carbons and Y denotes the total number of double bonds in acyl chains. Data are presented as means ± standard errors (SE); * *p* < 0.05; ** *p* < 0.01; *** *p* < 0.001, Student’s t-test.

To define relationships between cold acclimation and thermoregulatory behaviour in *Drosophila*, temperature preference in third instar larvae of *D. melanogaster* was evaluated using a thermal gradient plate after incubation at 25 °C or 18 °C for 1 day. Cold exposure significantly altered average preferred temperature from 22.5 °C ± 0.2 °C to 20.7 °C ± 0.3 °C (Fig. 1a,b and Supplementary Table S1), indicating that thermoregulatory behaviour is affected by environmental temperature. In subsequent experiments, we assessed the effects of cold exposure on fatty acid compositions of phospholipids using liquid chromatography-tandem mass spectrometry (LC–MS/MS) (Fig. 1c,d). The proportions of phosphatidylcholine (PC) and phosphatidylethanolamine (PE) molecules with ≤ one double bond in their acyl chains [PC (28:0), PC (30:0), PC (28:1), PC (30:1), PC (32:1), PC (34:1), PC (36:1), PE (30:0), PE (34:1) and PE (36:1)] were significantly decreased in cold-exposed larvae, with the exception of PE (32:1). In contrast, the proportions of PC and PE molecules with ≥ two double bonds in their acyl chains [PC (30:2), PC (32:2), PC (34:3), PC (36:4), PE (34:2) and PE (36:3)] were present at significantly increased in cold-exposed larvae. To determine whether PC and PE molecules with two or three double bonds includes PUFAs in their acyl chains, we performed product ion scan analyses of PC (32:2), PE (34:2) and PC (34:3) using LC–MS/MS (Supplementary Fig. S1). PC (32:2) yielded a product ion that coincided with C16:1 (*n*-7) (Supplementary Fig. S1a,b), but PE (34:2) and PC (34:3) yielded a product ion of C18:2 (*n*-6) (Supplementary Fig. S1c–f). The detected C18:2 (*n*-6) is presumed to be derived from their diet, because flies cannot synthesize C18:2 (*n*-6). In contrast, the product ion scan analysis of PC (32:1), PC (34:1) and PE (34:1), whose proportions were decreased in cold-exposed larvae, yielded mainly product ions of C16:1 (*n*-7), C18:1 (*n*-9) and C16:0 (Supplementary Fig. S2a–f). Hence, we hypothesised that cold exposure increases the presence of PUFA-containing phospholipids that promote thermoregulatory cold preference in *D. melanogaster* because C18:2 (*n*-6)-containing phospholipids were accumulated following cold exposure.

### Establishment of transgenic flies expressing Δ12 fatty acid desaturase

**Figure 2:**
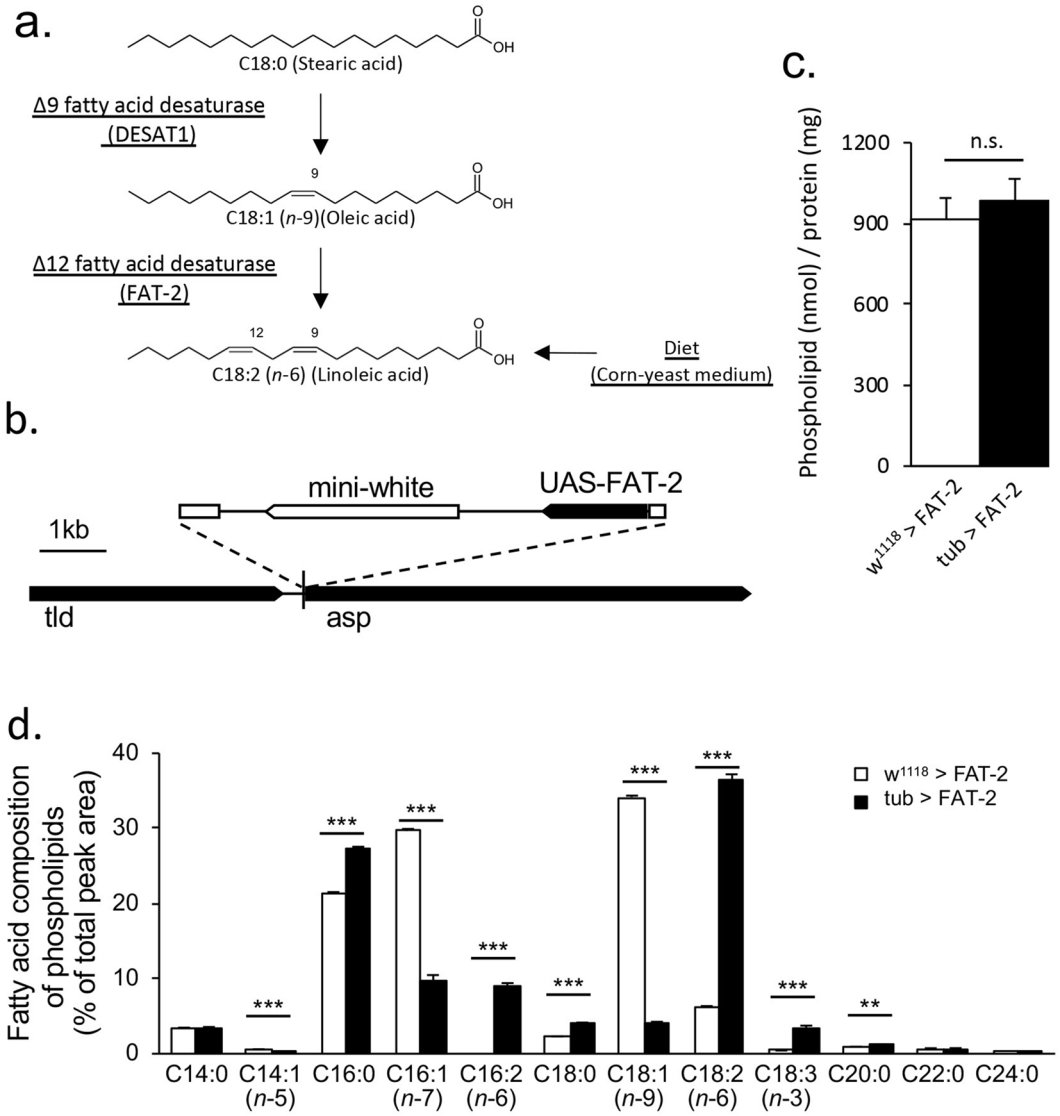
Establishment of transgenic flies expressing *Caenorhabditis elegans* Δ12 fatty acid desaturase (FAT-2). (**a**) Schematics overview of FAT-2-mediated C18:2 (*n*-6) synthesis in FAT-2-expressing *Drosophila.* (**b**) The genomic structure is depicted in the cartoon. A P-element including upstream activating sequence (UAS)-FAT-2 was inserted into the chromosome 3R between the genes *tolloid* (*tld*) and *abnormal spindle* (*asp*). (**c**) The amounts of the total phospholipids in control larvae (w^1118^ > FAT-2) and larvae ubiquitously expressing FAT-2 (tub > FAT-2) (n = 3, Student’s t-test). (**d**) The fatty acid compositions of phospholipids in control (w^1118^ > FAT-2, white bar; n = 3) and larvae ubiquitously expressing FAT-2 (tub > FAT-2, black bar; n = 3) were analysed using GC-FID. Data are presented as means ± SE; ** *p* < 0.01; *** *p* < 0.001, Student’s t-test.

To determine relationships between tissue-specific changes in lipid profiles and thermoregulatory behaviour, we employed the *Drosophila* GAL4/UAS system^38^ to modulate PUFA contents in a tissue-specific manner. Wild-type *D. melanogaster* only expresses Δ9 fatty acid desaturase and hence cannot produce PUFAs. Unlike *Drosophila*, *C. elegans* expresses Δ12 fatty acid desaturase (*fat-2*), which converts mono-unsaturated fatty acids to PUFAs such as C18:2 (*n*-6) and C18:3 (*n*-3)^14,25^ (Fig. 2a). In the present transgenic *D. melanogaster*, the UAS-*fat-2* construct was introduced using P-element-mediated transformation. The transgene was integrated between the two protein-encoded genes, namely, *tolloid* (*tld*) and *abnormal spindle* (*asp*), in chromosome 3 (Fig. 2b). To confirm functional expression of FAT-2 from the introduced GAL4/UAS system, phospholipids were extracted from third instar larvae ectopically expressing FAT-2 and fatty acid compositions were measured. The amount of the total phospholipids was not affected by the expression of FAT-2 (Fig. 2c). In the control strain (w^1118^ > FAT-2), C16:0, C16:1 (*n*-7) and C18:1 (*n*-9) were the most abundant fatty acids, and C18:2 (*n*-6) was only present in 6.3% of all phospholipid acyl chains. Ubiquitous expression of FAT-2 using the tub-GAL4 driver increased the proportion of C18:2 (*n*-6) to 36.4% and hexadecadienoic acid [C16:2 (*n*-6)] to 9.0% of all acyl chains of phospholipids. The proportions of C18:3 (*n*-3) was also increased in FAT-2-expressing larvae (Fig. 2d). This confirms that the introduced FAT-2 enzyme has Δ12/Δ15 desaturase activity.

### Effects of tissue-specific FAT-2 expression on lipid composition

**Figure 3:**
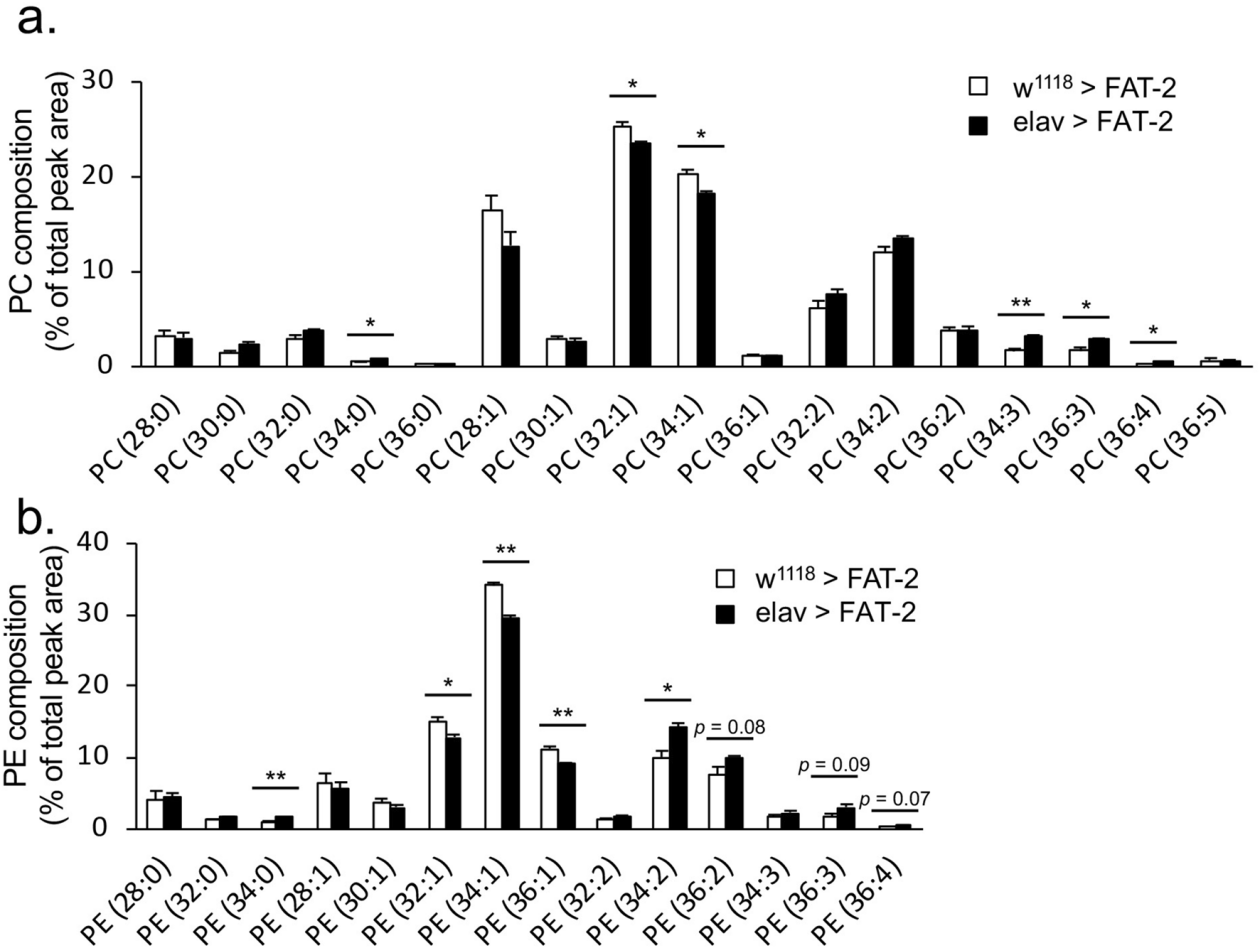
Changes in lipid compositions in CNS following neuron-specific expression of FAT-2. PC (**a**) and PE (**b**) molecules of CNS (brain and ventral nerve cord) in third instar larvae of control (w^1118^ > FAT-2, white bar, n = 3) and neuron-specific FAT-2-expressing specimens (elav > FAT-2, black bar, n = 3) were analysed using LC–MS/MS. Tissues were dissected from five third instar larvae. Data are presented as means ± SE; *p < 0.05; **p < 0.01, Student’s t-test.

To evaluate the effects of FAT-2 expression in a tissue-specific manner, we induced its expression by a variety of tissue-specific GAL4 drivers. The larvae expressing FAT-2 driven by r4-GAL4 (fat body) and Myo31DF-GAL4 (gut) showed that the proportions of C18:2 (*n*-6) in acyl chains of whole-body phospholipids were increased by up to 23.1% and 22.7%, respectively (Supplementary Fig. S3). In contrast, the FAT-2 expression driven by elav-GAL4 (neuron) did not significantly affect the proportion of C18:2 (*n*-6) in acyl chains of whole-body phospholipids. To determine why the proportion of C18:2 (*n*-6) in whole-body phospholipids was not affected by elav > FAT-2, we analysed lipids from central nervous system (CNS; brain and ventral nerve cord) using LC–MS/MS (Fig. 3a,b). In larvae expressing FAT-2 in neurons (elav > FAT-2), the proportions of phospholipids containing at least one PUFA [PC (34:3), PC (36:3), PC (36:4) and PE (34:2)] were significantly increased, and the proportions of phospholipids containing one mono-unsaturated fatty acid [PC (32:1), PC (34:1), PE (32:1), PE (34:1) and PE (36:1)] were significantly decreased in CNS. To further analyse phospholipid fatty acid compositions, we performed product ion scan analyses of PC (34:1), PC (34:2), PC (34:3), PC (36:2), PC (36:3), PE (34:1), PE (34:2), PE (34:3), PE (36:2) and PE (36:3) (Supplementary Fig. S4 and S5). In PC (34:1) (Supplementary Fig. S4a,b) and PE (34:1) (Supplementary Fig. S5a,b) whose proportions were decreased in the CNS of elav > FAT-2 larvae, product ions coinciding with C16:0, C16:1 (*n*-7), C18:0 and C18:1 (*n*-9) were detected. Conversely, ion fragments coinciding with C18:2 (*n*-6) were detected in PC (34:3) (Supplementary Fig. S4e,f), PC (36:3) (Supplementary Fig. S4i,j) and PE (34:2) (Supplementary Fig. S5c,d) whose proportions were increased in the CNS of elav > FAT-2 larvae. Although the proportions of PC (34:2) (Supplementary Fig. S4c,d), PC (36:2) (Supplementary Fig. S4g,h) and PE (36:2) (Supplementary Fig. S5g,h) were not significantly increased in CNS of elav > FAT-2 larvae (Fig. 3a,b), the proportions of C18:2 (*n*-6)-containing species were increased in these phospholipids.

### Effects of tissue-specific expression of FAT-2 on thermoregulatory behaviour

**Figure 4:**
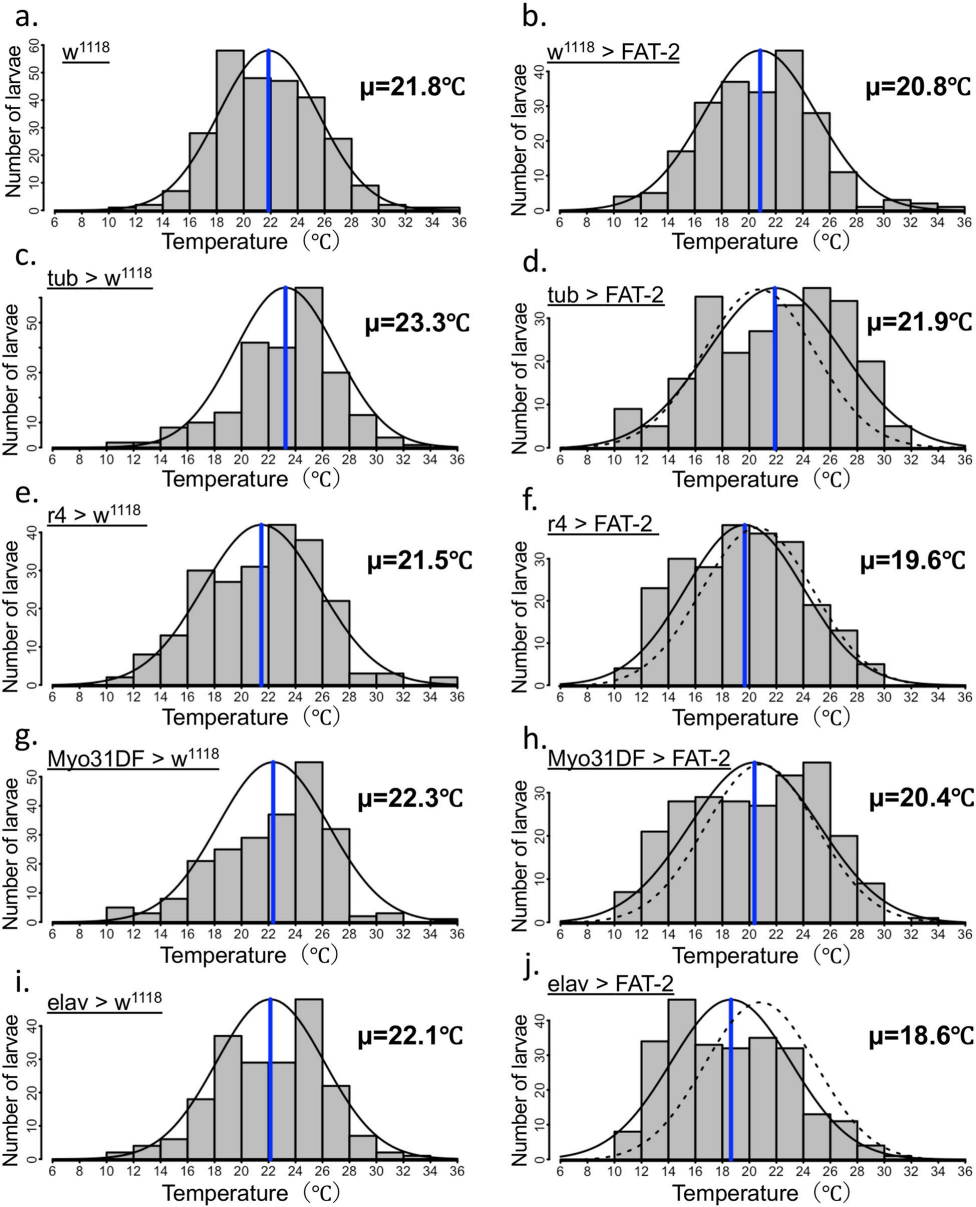
Temperature preference of third instar larvae expressing FAT-2 under the control of various tissue-specific GAL4 drivers. The histogram shows distributions of third instar larvae on the thermal gradient plate. The distribution curve is denoted by a black solid line and the average temperature preference is shown as a blue vertical line. The dotted curve represents the distribution of the control (w^1118^ > FAT-2). The temperature preference of third instar larvae expressing FAT-2 under the control of tissue-specific GAL4 drivers (tub-GAL4, r4-GAL4, Myo31DF-GAL4 and elav-GAL4) (n = 6) (**d**, **f**, **h** and **j**, respectively). As control, the temperature preference of third instar larvae in the w^1118^ strain crossed with the w^1118^ (n = 7) (**a**), tissue-specific GAL4 drivers (tub-GAL4, r4-GAL4, Myo31DF-GAL4 and elav-GAL4) (n = 6) (**c**, **e**, **g** and **i**, respectively) and UAS-FAT-2 strain (n = 6) (**b**) was analysed. The numerical analyses of data are also shown in Supplementary Table S2.

Although ubiquitous expression of FAT-2 (tub-GAL4) did not affect the temperature preference of larvae (Fig. 4b–d, and Supplementary Table S2), the preferred temperature of larvae was significantly decreased by the tissue-specific expression of FAT-2 in fat bodies (r4-GAL4) (Fig. 4f and Supplementary Table S2), gut tissues (Myo31DF-GAL4) (Fig. 4h and Supplementary Table S2) and neurons (elav-GAL4) (Fig. 4j and Supplementary Table S2) compared with the w^1118^ strain crossed with the tissue-specific GAL-4 driver (Fig. 4e,g,i and Supplementary Table S2). Compared with the control strain (w^1118^) (Fig. 4a), statistical significant changes in the preferred temperature were also observed in r4 > FAT-2 (Fig. 4f and Supplementary Table S2) and elav > FAT-2 (Fig. 4j and Supplementary Table S2). Among them, the average preferred temperature in elav > FAT-2 (18.6 °C ± 0.3 °C) (Fig. 4j and Supplementary Table S2) was remarkably decreased by 2.2 °C and 3.5 °C compared with the w^1118^ > FAT-2 (20.8 °C ± 0.3 °C) (Fig. 4b and Supplementary Table S2) and elav crossed with the w^1118^ (22.1 °C ± 0.3 °C) (Fig. 4i and Supplementary Table S2), respectively. Given that the greatest change in temperature preference was induced by neuron-specific expression of FAT-2, we further analysed the role of neuronal PUFAs in the regulation of thermoregulatory behaviour.

To determine whether the thermoregulatory behaviour is also affected by the expression of the desaturase with distinct substrate preference, we overexpressed the native *D. melanogaster* Δ9 fatty acid desaturase DESAT1 that produces mono-unsaturated fatty acids. We established a UAS-DESAT1 transgenic strain using the same methods as that of the generation of a FAT-2 transgenic strain (see “Methods”). In contrast to FAT-2 overexpression, no significant difference was observed in preferred temperature between control (elav > w^1118^) (22.9 °C ± 0.3 °C) (Supplementary Fig. S6a and Supplementary Table S3) and neuronal DESAT1-overexpressed larvae (elav > DESAT1) (22.4 °C ± 0.3 °C) (Supplementary Fig. S6b and Supplementary Table S3). These results suggest that the production of PUFAs, rather than mono-unsaturated fatty acids, in neurons contribute to the regulation of thermoregulatory behaviour in *Drosophila*.

### Energy metabolism is unchanged by neuronal expression of FAT-2

**Figure 5:**
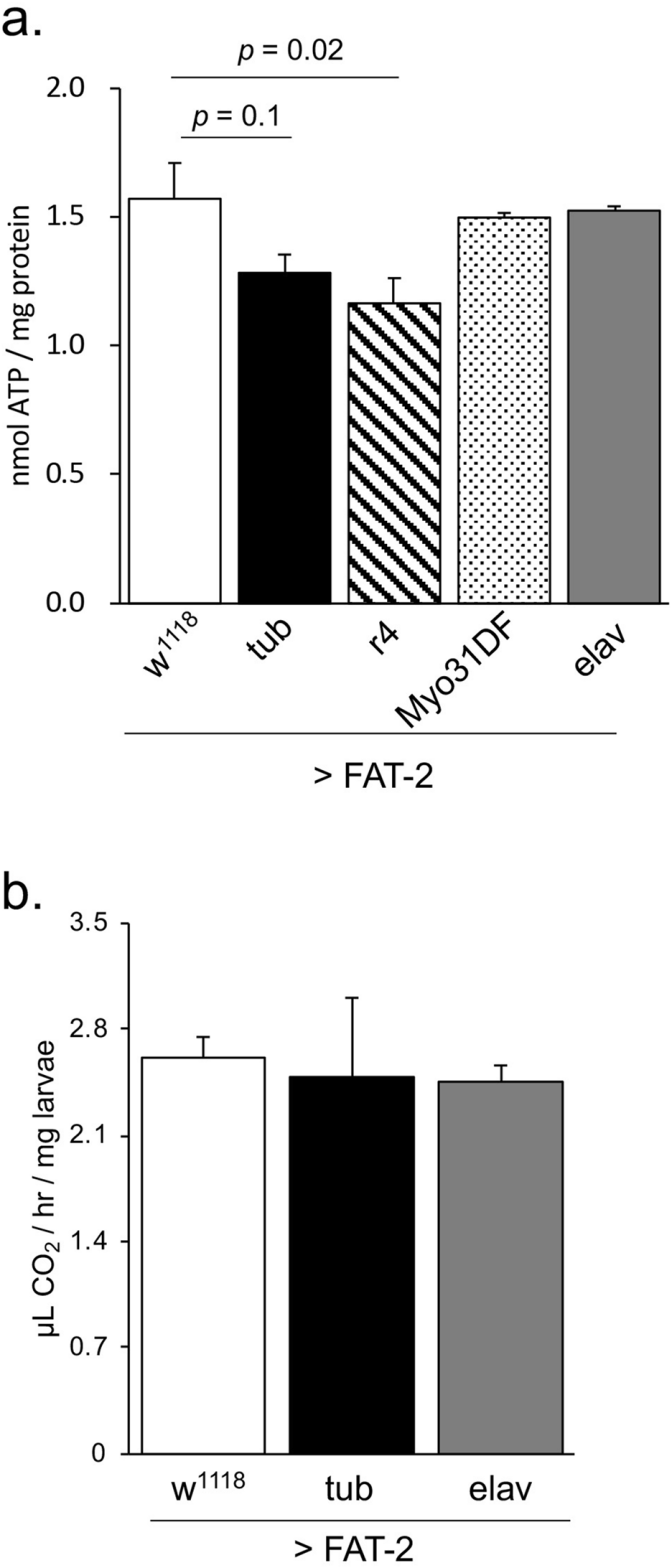
Effect of FAT-2 expression on the energy metabolism in third instar larvae. (**a**) ATP concentrations in the whole bodies of third instar larvae expressing FAT-2 in different tissues. Data are presented as means ± SE. Statistical analyses were performed using Dunnett’s test. (**b**) Metabolic rates of third instar larvae from control flies (w^1118^ > FAT-2, white bar, n = 3) and flies expressing FAT-2 in whole bodies (tub > FAT-2, black bars, n = 3) and neurons (elav > FAT-2, grey bar, n = 3). No significant differences were observed among samples using Tukey–HSD test. Data are presented as means ± SE.

In a previous study, we showed that the cryophilic mutant fly, *atsugari*, had increased energy metabolism^34^. Thus, to clarify the roles of energy metabolism in the phenotypes associated with FAT-2 expression, we measured adenosine triphosphate (ATP) concentrations and metabolic rates in FAT-2-expressing larvae. ATP concentrations were significantly decreased in larvae expressing FAT-2 in fat bodies, but were not affected by ubiquitous and neuron- or gut-specific expression of FAT-2 (Fig. 5a). Similarly, metabolic rates of neuronal or ubiquitous FAT-2-expressing larvae were comparable to those of control larvae (Fig. 5b). These results suggest that changes in the temperature preference of elav > FAT-2 larvae is likely not related to alterations of energy metabolism.

### FAT-2 expression affected TRPA1-expressing neuron-mediated thermoregulatory behaviour

**Figure 6:**
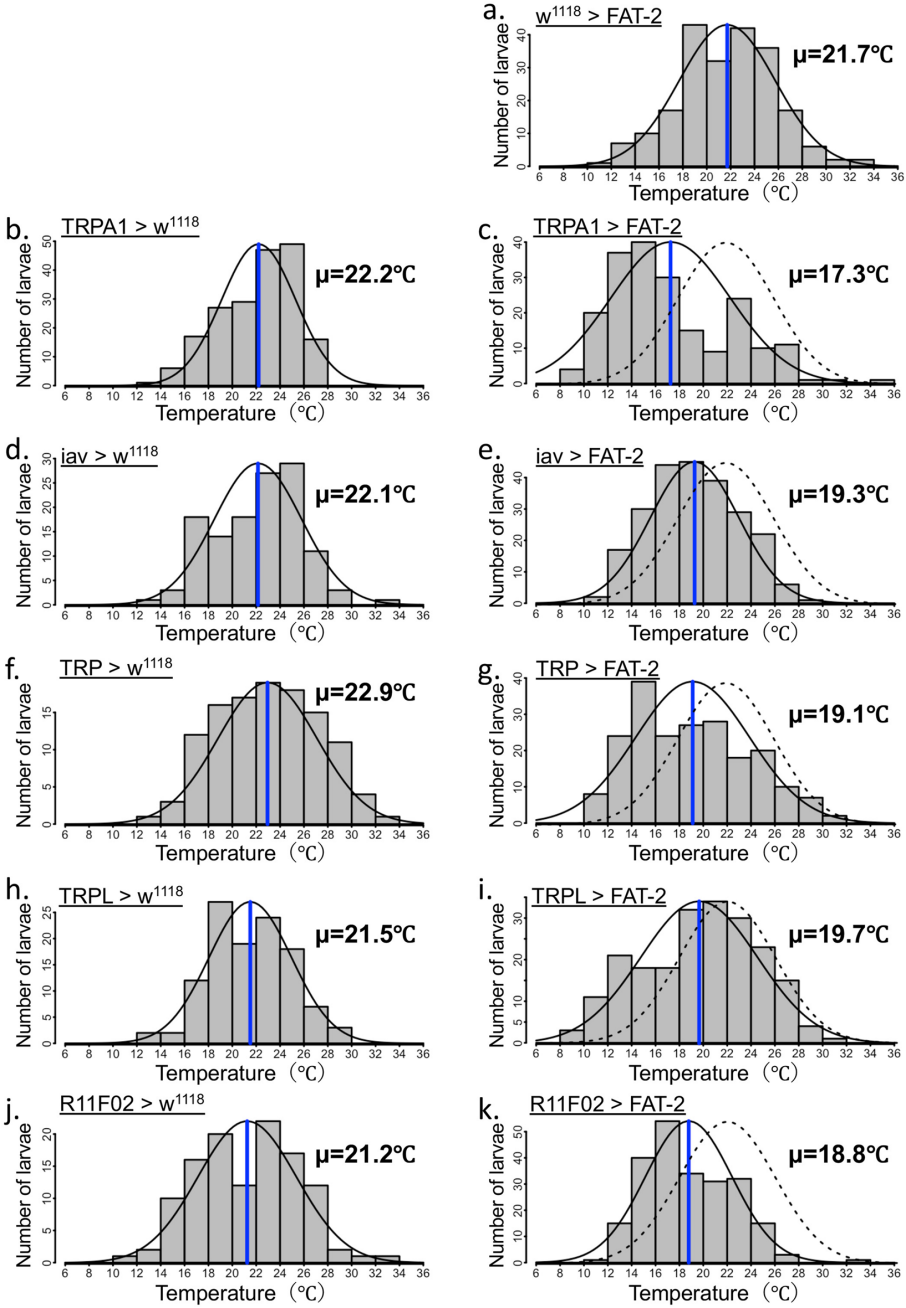
Ectopic expression of FAT-2 in thermoreceptor-expressing cells alters temperature preference of third instar larvae. The histogram shows distributions of third instar larvae on the thermal gradient plate. The distribution curve is denoted by a black solid line and the average temperature preference is shown as a blue vertical line. The dotted curve represents the distribution of the control (w^1118^ > FAT-2). The temperature preference of third instar larvae expressing FAT-2 under the control of various neuron-specific GAL4 drivers (TRPA1-GAL4, iav-GAL4, TRP-GAL4, TRPL-GAL4 and R11F02-GAL4) (n = 6) (**c**, **e**, **g**, **i** and **k**, respectively). As control, the temperature preference of third instar larvae in w^1118^ strain crossed with various neuron-specific GAL4 drivers (TRPA1-GAL4, iav-GAL4, TRP-GAL4, TRPL-GAL4 and R11F02-GAL4) (n = 6) (**b**, **d**, **f**, **h** and **j**, respectively) and UAS-FAT-2 strain (n = 6) (**a**) was analysed. The numerical analyses of data are also shown in Supplementary Table S4.

**Figure 7:**
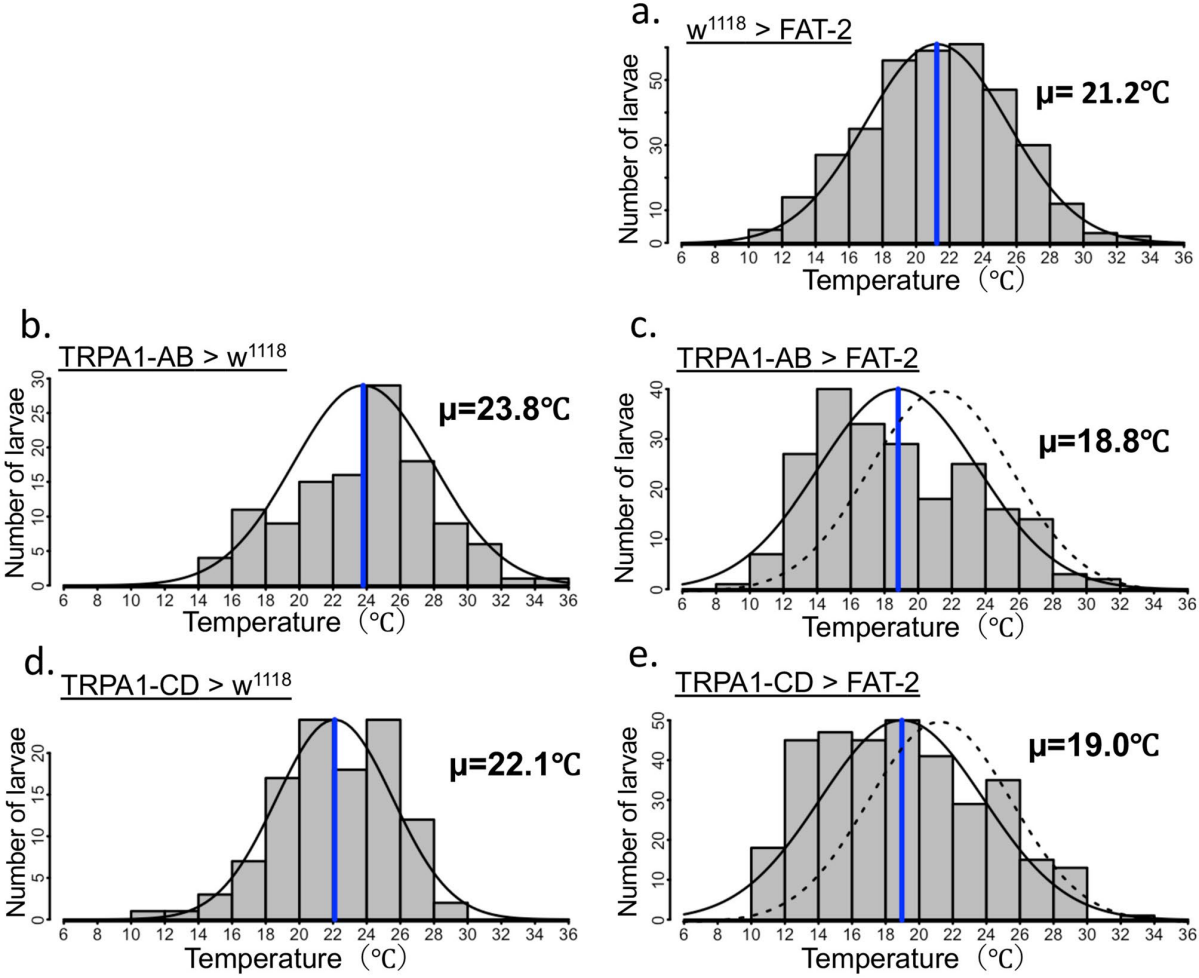
Temperature preference of third instar larvae expressing FAT-2 in distinct TRPA1-expressing neurons. The histogram shows distributions of third instar larvae on the thermal gradient plate. The distribution curve is denoted by a black solid line and the average temperature preference is shown as a blue vertical line. The dotted curve represents the distribution of control (w^1118^ > FAT-2) flies. The temperature preference of third instar larvae expressing FAT-2 under the control of TRPA1-expressing neuron-specific GAL4 drivers (TRPA1-AB-GAL4 and TRPA1-CD-GAL4) (n = 6) (**c** and **e**, respectively). As control, the temperature preference of third instar larvae in w^1118^ strain crossed with TRPA1-expressing neuron-specific GAL4 drivers (TRPA1-AB-GAL4 and TRPA1-CD-GAL4) (n = 3) (**b** and **d**, respectively) and UAS-FAT-2 strain (n = 9) (**a**) was analysed. The numerical analyses of data are also shown in Supplementary Table S5.

**Figure 8:**
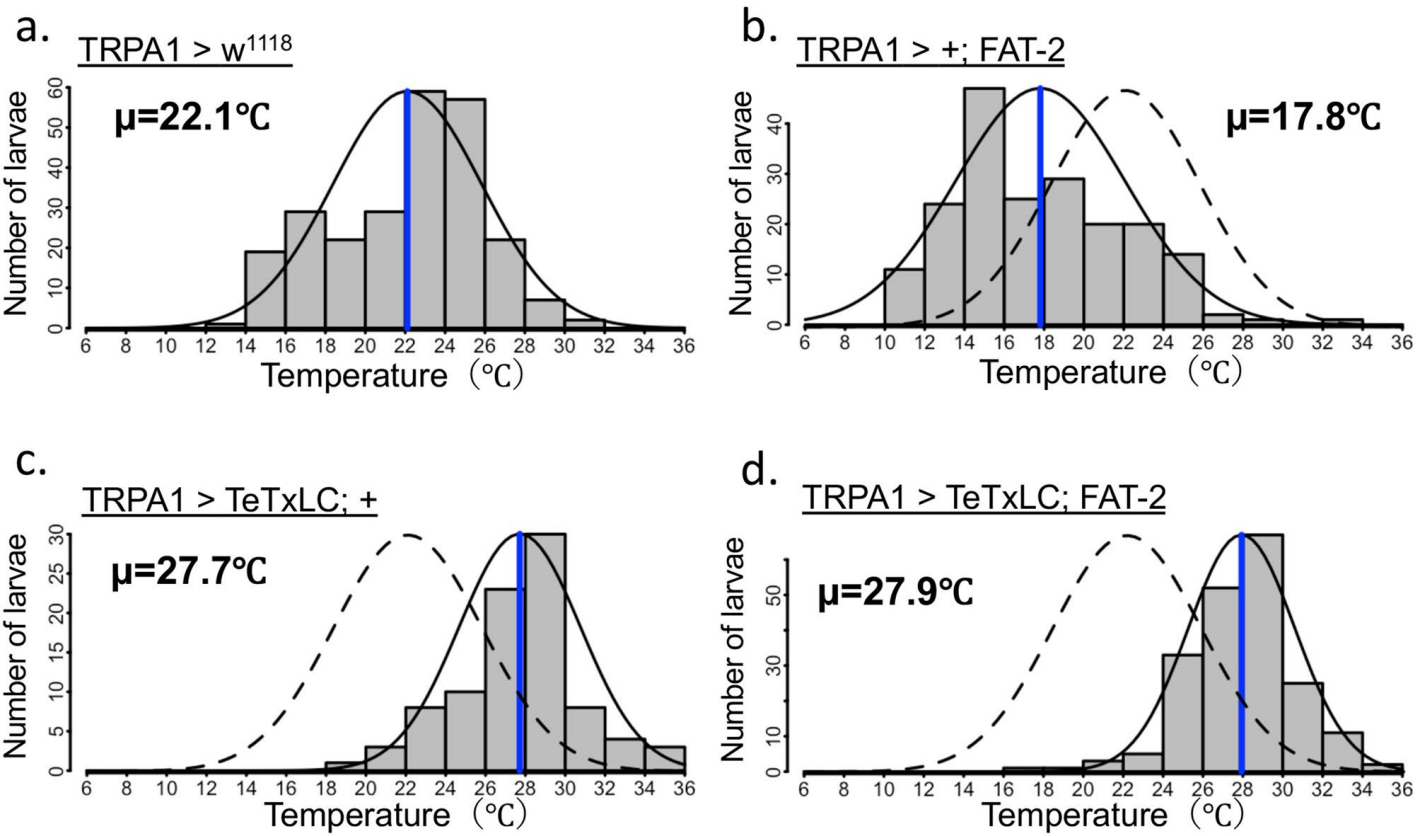
Temperature preference of third instar larvae expressing FAT-2 and TeTxLC in TRPA1-expressing neurons. The histogram shows the distributions of third instar larvae on the thermal gradient plate. The distribution curve is denoted by a black solid line and the average temperature preference is shown as a blue vertical line. The dotted curve represents the distribution of control (w^1118^ > FAT-2) flies. Temperature preference in third instar larvae of control (TRPA1-GAL4 > w^1118^) (n = 6) (**a**), TRPA1-GAL4 > FAT-2 (n = 6) (**b**), TRPA1-GAL4 > TeTxLC (n = 8) (**c**) and TRPA1-GAL4 > TeTxLC; FAT-2 (n = 8) (**d**). The numerical analyses of data are also shown in Supplementary Table S6.

Because thermoregulatory behaviour in *D. melanogaster* is closely associated with thermosensation^39^, neuron-specific expression of FAT-2 may affect thermosensory neuron activities and influence temperature preference. To identify thermosensory neurons that are responsible for FAT-2-induced changes in temperature preference, FAT-2 was expressed in various thermoreceptor-expressing neurons, which reportedly determine temperature preference at ambient temperatures. We used transient receptor potential A1 (TRPA1)-GAL4 as a driver of warm sensory neurons^40^ and iav-GAL4^41^, R11F02-GAL4^42,43^, TRP-GAL4^44^ and TRPL-GAL4^44^ as drivers of cold sensory neurons. Compared with control larvae (w^1118^ > FAT-2) (Fig. 6a and Supplementary Table S4) or thermoreceptor-specific GAL4 drivers crossed with w^1118^ (Fig. 6b,d,f,h,j and Supplementary Table S4), the preferred temperature was significantly decreased in larvae expressing FAT-2 induced by all thermoreceptor-specific GAL4 drivers (Fig. 6c,e,g,i,k and Supplementary Table S4). Although decreases in preferred temperature was observed with all GAL4 constructs (Fig. 6a–k and Supplementary Table S4), the most drastic shift to low temperature was induced by FAT-2 expression in TRPA1-expressing neurons (Fig. 6c and Supplementary Table S4). TRPA1 has at least four isoforms and is transcribed from two distinct promoters^45^. TRPA1-A and TRPA1-B were expressed in the brain using the first promoter, whereas TRPA1-C and TRPA1-D were expressed mainly in multidendritic class IV neurons using the second promoter. FAT-2 expression under the control of either TRPA1-AB-GAL4 or TRPA1-CD-GAL4 caused significant decrease in preferred temperature (Fig. 7a–e and Supplementary Table S5). Next, to investigate whether TRPA1-expressing neurons are important for FAT-2-induced changes in temperature preference, we recorded the temperature preference of larvae expressing tetanus toxin light chain (TeTxLC), an inhibitor of synaptic vesicle release^46^, with (Fig. 8b,d) or without (Fig. 8a,c) FAT-2 expression under the control of TRPA1-GAL4. Larvae expressing TeTxLC under the control of TRPA1-GAL4 preferred significantly higher temperature than control larvae, as previously reported^47^ (Fig. 8a,c and Supplementary Table S6). Moreover, temperature preference of TeTxLC-expressing larvae was not changed by additional expression of FAT-2 under the control of TRPA1-GAL4 (Fig. 8c,d and Supplementary Table S6). These results demonstrate that FAT-2 expression in TRPA1-expressing neurons changes the thermoregulatory behaviour of *D. melanogaster*.

### TRPA1-expressing neuron activity is affected by FAT-2 expression

**Figure 9:**
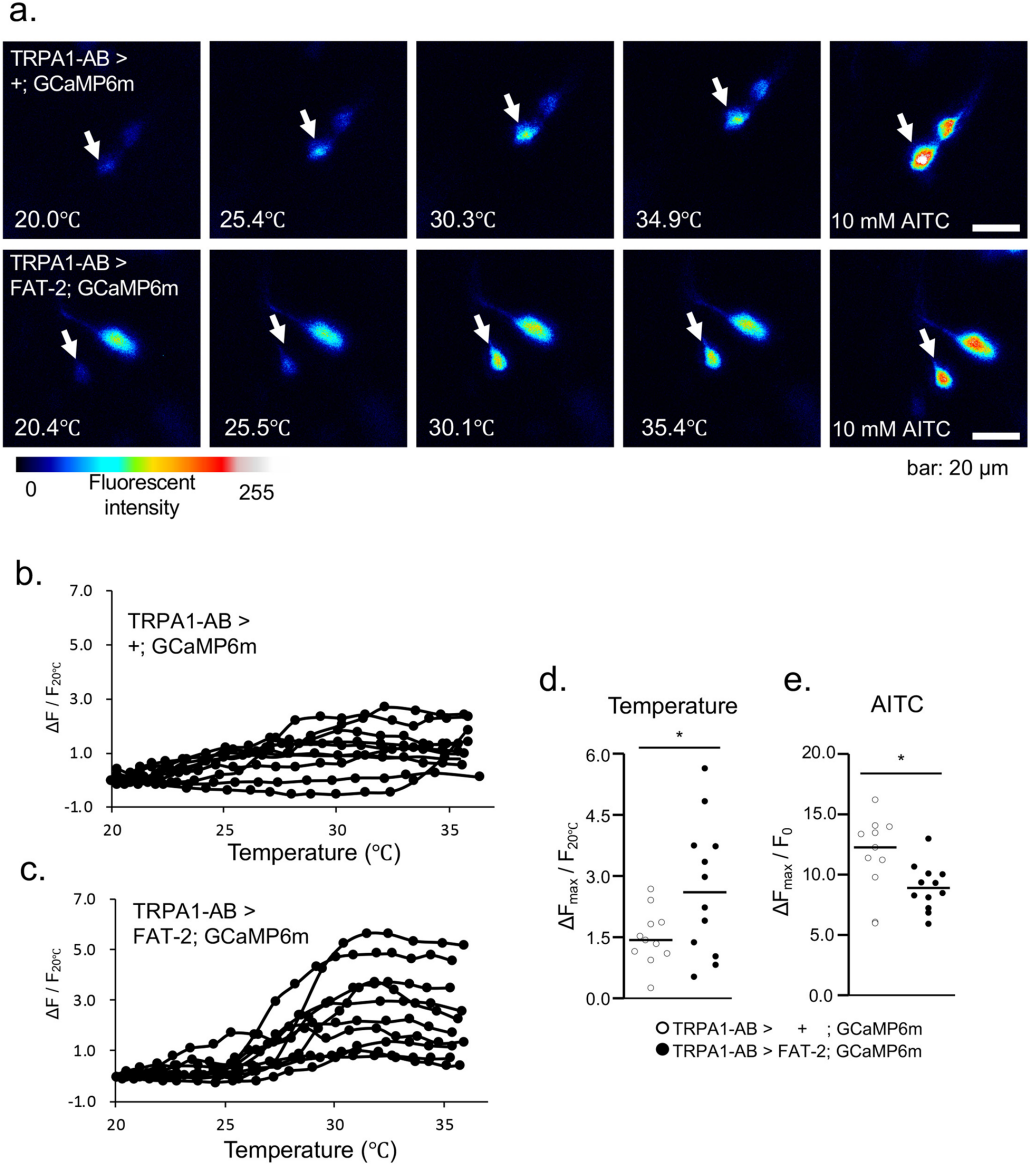
Ca^2+^ measurements in TRPA1-expressing neurons. (**a**) UAS-GCaMP6m was expressed under the control of TRPA1-AB-GAL4. The thermosensitive brain lateral posterior (BLP) neurons (white arrow) were activated by raising the temperature or using the TRPA1 activator allyl isothiocyanate (AITC) (10 mM) at 20 °C in the control (TRPA1-AB >  + ; GCaMP6m, upper row) and FAT-2-expressing (TRPA1-AB > FAT-2; GCaMP6m, lower row) neurons. Colours indicate fluorescence intensities in the 8-bit range. BLP neuron activity in TRPA1-AB >  + ; GCaMP6m (n = 11) (**b**) and TRPA1-AB > FAT-2; GCaMP6m (n = 12) (**c**) were quantified as ∆F/F_20°C_ and were plotted against temperature. (**d**) The dot plot of maximum activities following temperature activation in TRPA1-AB >  + ; GCaMP6m (white dot) and TRPA1-AB > FAT-2; GCaMP6m (black dot) neurons; bars indicate medium values. (**e**) AITC activation of BLP after temperature activation in TRPA1-AB >  + ; GCaMP6m (n = 11) (white dot) and TRPA1-AB > FAT-2; GCaMP6m (n = 12) (black dot) neurons. AITC activations were measured in the same neurons after temperature-induced activation. Activity was calculated as ∆F_max_/F_20°C_ or ∆F_max_/F_0_, where F_20°C_ and F_0_ were the fluorescent intensities at 20 °C and at the initial timepoint, respectively, and ∆F_max_ was calculated by subtracting F_20°C_ or F_0_ from maximum fluorescent intensities. Bars indicate medium values. P-values were calculated using Student’s t-tests (*p < 0.05).

To test whether TRPA1-expressing neuron activities are affected by FAT-2 expression, we performed Ca^2+^ imaging in neurons using UAS-GCaMP6m^48^. We dissected CNS from third instar larvae expressing GCaMP6m (UAS-GCaMP6m/ + ; TRPA1-AB-GAL4/ +) or both GCaMP6m and FAT-2 (UAS-GCaMP6m/ + ; TRPA1-AB-GAL4/UAS-FAT-2). Among TRPA1-expressing neurons, responses in thermosensitive brain lateral posterior (BLP) neurons^49^ were recorded at temperatures ranging from 20 to 35 °C (Fig. 9a). Temperature-sensitive responses were observed in both control and FAT-2-expressing neurons, yet maximal intensities were greater in FAT-2-expressing neurons (Fig. 9b–d). To evaluate temperature-independent activities of TRPA1-expressing neurons, we measured responses to the TRPA1 activator allyl isothiocyanate (AITC). The responses to AITC at 20 °C were not increased in FAT-2-expressing neurons (Fig. 9e), suggesting that the increase in temperature-dependent maximal activity of FAT-2-expressing neurons was not caused by temperature-independent effects, such as increased TRPA1 expression levels.

To analyse the effects of C18:2 (*n*-6) on TRPA1 channel activities, we performed cell-based assays (Supplementary Fig. S7 and S8). Incubation of *Drosophila* S2 cells with bovine serum albumin (BSA)-bound fatty acids for 6 h increased the proportion of added fatty acids in acyl chains of phospholipids^50^. In analyses of Ca^2+^ imaging in TRPA1-transfected S2 cells, temperature-induced maximal activity of TRPA1 was increased by 6 h treatments with C18:2 (*n*-6) (Supplementary Fig. S7b,d) but not with C18:1 (*n*-9) (Supplementary Fig. S7c,d). Moreover, following perfusions with 100 μM non-esterified C18:2 (*n*-6) or C18:1 (*n*-9) (Supplementary Fig. S8), small but significant increases in intracellular Ca^2+^ concentrations were observed (Supplementary Fig. S8b–d). However, the resulting TRPA1 activities induced by perfusion of fatty acids (Supplementary Fig. S8b,c) were only 1/10 compared to those induced by temperature changes (Supplementary Fig. S7a), suggesting that TRPA1 is not activated by non-esterified C18:2 (*n*-6).

These results indicate that increases in C18:2 (*n*-6)-containing phospholipids in TRPA1-expressing neurons enhance their sensitivity to warm temperature, resulting in changed thermoregulatory behaviour in *D. melanogaster*.

## Discussion

In this study, we established a transgenic *Drosophila* line of the *C. elegans* Δ12 fatty acid desaturase FAT-2, which was functionally expressed in *Drosophila* (Fig. 2d). The desaturation of fatty acids is performed by a multi-enzyme complex comprising cytochrome b5, cytochrome b5 reductase and fatty acid desaturase. FAT-2 carries the putative cytochrome b5 domain but requires the specific cytochrome b5 reductase HPO-19 and T05H4.4 for activation in *C. elegans*^51^. In *D. melanogaster*, the putative cytochrome 5b reductase CG5946 (NP_729751.1) has 59% and 55% amino acid sequence identity with HPO-19 (NP_504638.1) and T05H4.4 (NP_504639.1), respectively, and likely donates electrons for FAT-2 activity.

Dietary PUFAs are known to affect thermoregulation in animals, such as lizards^52,53^ and birds^54^. In addition, the dietary supplementation with C18:2 (*n*-6) has been reported to affect the visual system^36^ and survival at cold temperatures in *Drosophila*^30^. However, the significant roles being played by the changes in lipid profiles of specific tissues remain unknown. In the present study, we employed the GAL4/UAS system to express FAT-2 in a tissue-specific manner and found that neuronal expression of FAT-2 confers the most significant decrease in preferred temperature in the third instar larvae of *Drosophila*. Since significant increases in the proportion of PUFA-containing phospholipids were observed in the CNS of the larvae expressing FAT-2, it is apparent that the modulation of lipid molecules in the neurons was sufficient to affect behavioral thermoregulation in *Drosophila*.

In *D. melanogaster*, various thermosensitive receptors have been identified using a combination of genetic approaches, electrophysiological techniques and analyses of temperature preference^55,56^. The TRPV family member inactive (IAV) is expressed in chordotonal organs of flies and is required for cold sensation^41^. Moreover, the TRPC family members TRP and TRPL are reportedly required for cold avoidance^44^. Channel proteins of other protein families have also been associated with thermoregulatory behaviours in *D. melanogaster* larvae. Among these, the ionotropic receptors IR21a and IR25a are expressed in cold-sensitive dorsal organ neurons of larvae and are required for cold avoidance^43^. TRP channel family members are the most extensively studied thermoreceptor members and function as sensors for various environmental cues, including chemical and physical stimuli^57–59^. Among them, the TRPA1 channel is well known for its roles in the detection of noxious chemicals and unfavorable temperatures in various animals^60^. Although the thermosensitivity of TRPA1 remains controversial in mammals, the *Drosophila* TRPA1 channel is demonstrably activated by elevated temperature and by noxious chemicals^60,61^. In *Drosophila*, TRPA1 was identified as the receptor that controls thermoregulatory behaviour^40^. Moreover, TRPA1 is expressed in anterior cell neurons of adult *D. melanogaster*, and TRPA1-deficient *D. melanogaster* show decreased aversion to warm temperatures^62^. TRPA1 is also important for the temperature preference^47,63^ or warm temperature-induced rolling behaviour^49^ of *D. melanogaster* larvae. We demonstrated that responses of TRPA1-expressing neurons to warm temperatures were significantly increased by FAT-2 expression (Fig. 9c), suggesting that PUFAs, such as C18:2 (*n*-6), affects thermoregulatory behaviour by regulating the activities of TRPA1-expressing neurons. Recently, Umezaki et al. showed that starvation caused a cold preference and a change in the response to temperature in TRPA1-expressing neurons (AC neuron) via modulation of the insulin/insulin-like growth factor signaling pathway in adult *Drosophila*^39^. Although the process from temperature reception to thermoregulatory behaviour has not been completely revealed, it is apparent that the modulation of the activity of TRPA1-expressing neurons is a crucial mechanism for the modulation of thermoregulatory behaviour in *Drosophila* in different conditions.

FAT-2-mediated decreases in preferred temperature were also observed in larvae expressing FAT-2 under control of other GAL4-thermoreceptor constructs that are associated with cold sensation (Fig. 6 and Supplementary Table S4). Previous studies showed that the TRP channel activities were modulated by lipid molecules^64^. In particular, C18:2 (*n*-6) reportedly activated the TRPL in *Drosophila*^35,65^, and inhibit the response to menthol in mammalian TRPM8 and response to capsaicin in mammalian TRPV1^66^. Although the effects of PUFAs on the cold sensation by iav and ionotropic receptors have not been reported, the C18:2 (*n*-6) production by FAT-2 in cold-sensitive neurons may desensitize or inhibit thermoreceptors that are involved in the cold sensations. The evaluation of the effects of C18:2 (*n*-6) on each predicted cold channel using the cell-based assay that was used in this study for TRPA1 analysis (Supplementary Fig. S7) may reveal the actual regulatory functions of C18:2 (*n*-6) with respect to cold receptors.

Motter et al. expressed rat TRPA1 in HEK293T cells and showed strong activation following treatments with arachidonic acid [C20:4 (*n-*6)], eicosapentaenoic acid [C20:5 (*n-*3)] and docosahexaenoic acid [C22:6 (*n-*3)] but only weak activation by C18:2 (*n-*6)^67^. Yet in their experiments, *D. melanogaster* TRPA1 was not activated by C22:6 (*n-*3). These investigators also showed that activation of TRPA1 by PUFAs does not involve the known ligand-binding domains, suggesting that other transmembrane or intracellular domains mediate direct interactions with PUFAs. In our study, non-esterified C18:2 (*n-*6) did not affect the function of *D. melanogaster* TRPA1 as an activator (Supplementary Fig. S8), indicating that C18:2 (*n*-6) is not likely to be a ligand of *Drosophila* TRPA1. We also showed that the responses to AITC were not increased in FAT-2-expressing neurons (Fig. 9e). This suggests that the increased temperature-dependent activities in FAT-2-expressing neurons were not caused by temperature-independent events, such as increased expression of TRPA1. Several TRP channels carry phosphatidylinositol-binding domains that are required for regulation of channel activity^68^. Structural analysis also demonstrated the presence of a phospholipid-binding motif in TRPA1, suggesting that TRPA1 is regulated by direct interactions with phospholipids^69^. In another study, TRP channels activities were highly sensitive to changes in the physicochemical properties of bilayer membranes, such as membrane tension^70^. Moreover, the domains required for thermal activation of TRPA1 were found in pore regions^71^ or in N-terminal regions upstream of ankyrin repeats^45,72^. These domains were distinct from those related to activation by AITC^73^. Thus, PUFA-containing phospholipids may affect the thermosensitive domains of TRPA1 through direct binding or the modulation of the physicochemical properties of the lipid membrane (Supplementary Fig. S9); however, further studies are required to elucidate the mechanisms involved at the molecular level.

We recently observed an accumulation of PUFA-containing phospholipids in the CNS of *D. melanogaster*^74^. PUFA-containing phospholipids are transported to the CNS through the receptor-mediated endocytosis of lipophorin^74^. Accordingly, we detected remarkably high expression levels of lipophorin receptors LpR1 and LpR2 in the CNS. From these observations, it is apparent that PUFAs are selectively transported to the CNS and might have specific functions in neurons. In the present study, we revealed that PUFAs in the phospholipid acyl chains in the thermosensor-expressing neurons affect the activity of thermosensory neurons and thermoregulatory behaviour. Further detailed analysis of lipophorin receptor expression in thermosensor-expressing neurons will elucidate the mechanisms of PUFA-mediated control of thermoregulatory behaviour at the molecular level. Moreover, novel physiological meanings and molecular mechanisms underlying the regulations of the contents and distribution of PUFA-containing lipid molecules will be revealed by tissue-specific expression of FAT-2 using a transgenic fly strain established in this study.

## Methods

### Fly strains

The fly strains r4-GAL4 (#33832), elav-GAL4 (#8760), TRPA1-GAL4 (#27593), TRP-GAL4 (#36359), TRPL-GAL4 (#52274), R11F02-GAL4 (#49828), iav-GAL4 (#52273), TRPA1-AB-GAL4 (#67131), TRPA1-CD-GAL4 (#67133), UAS-TeTxLC (#28838) and UAS-GCaMP6m (#42748) were obtained from the Bloomington Drosophila Stock Center (Indiana, USA). Myo31DF-GAL4 (#112001) and tub-GAL4 (#108074) strains were obtained from Kyoto stock center (DGRC) (Kyoto Institute of Technology, Japan). w^1118^ was provided from NIG-FLY stock center (National Institute of Genetics, Japan).

Fly stocks were raised on a corn/yeast/glucose medium containing 80 g brewer's yeast powder, 100 g glucose, 40 g cornmeal, and 7 g agar, 8.8 mL propionic acid, and 0.88 g butyl parahydroxybenzoate per 1 L water. Fly stocks were cultured at 25 °C with a 12-h light/12-h dark cycle. The strain w^1118^ was used as a control. In cold acclimation assays, vials were placed in an incubator at 18 °C for 1 day (20–23 h).

### Establishment of UAS-FAT-2 and UAS-DESAT1 transgenic *Drosophila*

To generate UAS-FAT-2 transgenic *Drosophila*, *C. elegans fat-2* cDNA clone yk714b3 was obtained from the EST library (https://nematode.lab.nig.ac.jp/db2/ShowCloneInfo.php?clone=714b3). The *fat-2* cDNA insert was amplified by PCR using primers 5′-GGAATTCATGACAATCGCTACAAAAGTGAAC -3′ and 5′- CCGCTCGAGGCAAAGGCTAAGAAGGCTCAA -3′. The PCR product was then digested with EcoRI and XhoI at the 5′ and 3′ terminals, respectively.

For generating UAS-DESAT1 transgenic *Drosophila*, the coding sequence of *D. melanogaster* DESAT1 was isolated from cDNA library of the fly strain of Canton-S as previously described^50^. The DESAT1 cDNA was amplified by PCR using primers 5′- CTGAAGTAAAACAGTTGTTGCAACATGC -3′ and 5′- CATGATTGGCCCTACGCTCAACCTGCCT -3′, and the resulting PCR product was cloned into a vector using the TA-cloning method. The cDNA insert was then digested with EcoRI and BglII at the 5′ and 3′ terminals, respectively.

The digested cDNA inserts were cloned into the pUAST vector^38^ at the corresponding cloning sites. The resulting constructs were subjected to P element-mediated transformation of the w^1118^ strain^75^. The integrated position of the transgene was determined through a sequence analysis of the franking genome.

### Temperature preference assays

Temperature preference of wandering third instar larvae was assayed as described previously^34^. Briefly, a temperature gradient was generated on an aluminium plate with a peltier device at both ends of the plate (DIA Medical System Co.). A glass plate covered with 2.2% agarose was placed on the aluminium plate. Subsequently, 30–40 larvae were placed on the position of the glass plate at 28 °C. Distributions of larvae were measured after 20 min.

### Lipid analysis

Lipids were extracted from homogenised samples using the Bligh and Dyer method^76^ and were then dissolved in chloroform. Phospholipids were fractionated from total lipid extracts using thin layer chromatography, using hexane/diethyl ether/acetic acid (60:40:1, v/v/v) as the solvent.

The amount of the total phospholipids was determined by inorganic phosphate quantification as previously described^77^ and normalised to protein contents that were measured using Pierce BCA Protein Assay Kits (Thermo Fisher, USA).

Lipid analyses using gas chromatography (GC) were performed as described previously^5,50^. The extracted phospholipids were incubated in a 5% hydrogen chloride/methanol solution (Nacalai Tesque, Japan) at 100 °C for 3 h. Fatty acid methyl esters were then analysed using GC-14A (Shimadzu, Kyoto, Japan) with a flame ionisation detector and Supelco Omegawax Capillary GC columns (0.25 μm, 30 m × 0.25 mm; Sigma-Aldrich, USA). The column temperature was held at 180 °C for 5 min, was ramped to 220 °C at 3 °C/min and then held for 7 min and was finally ramped to 240 °C at 3 °C/min and held for 15 min. Fatty acid peaks were identified using GC–MS.

LC–MS/MS analyses were performed using a high-performance liquid chromatography system LC-30AD (Shimadzu, Kyoto, Japan) coupled to a triple quadrupole mass spectrometer LC–MS-8040 (Shimadzu, Kyoto, Japan) that was equipped with an electrospray source as described previously^5,74^. The extracted phospholipids were separated using a Kinetex C8 column (2.6 μm, 2.1 × 150 mm) (Phenomenex, USA) with mobile phases comprising 10 mM ammonium formate in water (mobile phase A) and 10 mM ammonium formate in 2-propanol/acetonitrile/water (45/45/10) (mobile phase B). The gradient of mobile phase B was controlled by a pump, with an initial isocratic flow at 20% B for 1 min, a linear increase to 40% B over 1 min and then to 92.5% B with a curved gradient over 23 min, followed by a linear increase to 100% B over 1 min and 100% B for 4 min. The total flow rate was 0.3 mL/min, the column temperature was 45 °C, and the sampler temperature was set at 4 °C. Multiple reaction monitoring transitions were [M + H]^+^  → 184.0 for PC and [M + H]^+^  → [M + H–141.1]^+^ for PE. The fatty acid composition of PE and PC was determined by production scan analysis of [M–H]^−^ and [M + HCOO]^−^ as precursor ions, respectively.

### ATP measurements

ATP concentrations were measured as described previously^34^. Briefly, ten third instar larvae were homogenised in lysis buffer of an ATP Bioluminescence Assay Kit HS II (Roche, Switzerland) on ice and were then incubated at 72 °C for 15 min. Subsequently, homogenates were centrifuged at 15,000 rpm for 5 min and supernatants were centrifuged again. Supernatants were mixed with luciferase reagent and luciferase activity was quantified using an Infinite F200 pro (TECAN, Switzerland) instrument. ATP concentrations were normalised to protein concentrations that were measured using Pierce BCA Protein Assay Kits (Thermo Fisher, USA).

### Measurements of metabolic rates

Metabolic rates of *D. melanogaster* were measured according to CO_2_ production as described previously^34^. Briefly, ten third instar larvae were placed in a 1-mL plastic syringe containing a small piece of soda lime (Wako, Japan). A glass capillary was connected to one end of the syringe and a small amount of ink was placed at the end of the capillary. The syringe was then preincubated at 25 °C for 15 min and movements of ink were measured over 1 h. Volumes of CO_2_ produced were normalised to the body weights of ten larvae.

### Ca^2+^ imaging

To measure responses of TRPA1-expressing neurons, UAS-GCaMP6m was expressed using TRPA1-AB-GAL4. Brains were dissected from third instar larvae and were collected in recording buffer containing 5 mM TES, 10 mM HEPES, 120 mM NaCl, 3 mM KCl, 4 mM MgCl_2_, 2 mM CaCl_2_, 10 mM NaHCO_3_, 10 mM trehalose, 10 mM glucose and 10 mM sucrose (pH 7.25). Brain samples were placed on glass-bottomed dishes coated with poly-L-lysine, and the dishes were then filled with recording buffer and placed on a DTC-300C temperature controller (DIA Medical System Co., Tokyo, Japan). The setting temperature was increased from 19 to 40 °C, and the temperature near sample was recorded using a TA-29 thermistor (Warner instrument, USA). Ca^2+^ imaging was acquired using an inverted Zeiss LSM 800 confocal microscope equipped with a 10 × /0.25 objective and ZEN2.3 software (https://www.zeiss.com/microscopy/int/products/microscope-software/zen.html#modules). Increases in Ca^2+^ concentrations in BLP neurons were measured and activities were calculated as ∆F/F_20 °C_, where F_20 °C_ is the fluorescent intensity at 20 °C and ∆F is the change in fluorescence from F_20 °C_.

For cellular Ca^2+^ imaging, *Drosophila* S2 cells were maintained in Schneider’s *Drosophila* medium supplemented with 10% fetal bovine serum (FBS), 50-units/ml penicillin, and 50 μg/ml streptomycin at 25 °C. The TRPA1 (isoform A) expression vector was generated by cloning into the pAc5.1 plasmid and was subsequently transfected with the pCoBlast plasmid using TransFectin lipid reagent (Bio-Rad). Stable transformants were selected using 20 μg/ml blasticidin. Cells were incubated with 100 μΜ fatty acid [C18:1 (*n-*9) or C18:2 (*n-*6)]-BSA complex for 6 h as described previously^50^. Prior to Ca^2+^ measurements, cells were seeded on glass coverslips and were loaded with Fura2-AM (10 μM) (Dojindo, Japan) and probenecid (500 μM) in Schneider’s *Drosophila* medium for 60 min at 25 °C. After washing with recording buffer, coverslips were placed and slides were mounted in a perfusion chamber on a microscope (Axio-observer Z1, Carl-Zeiss, Germany). The recording buffer used in TRPA1-expressing neurons was supplemented with 500 μM probenecid and 1 mg/ml BSA. The temperature of the perfusion solution was controlled using a CL-100 bipolar temperature controller equipped with an SC-20 in-line solution heater/cooler (Warner Instruments, USA). Time-lapse images were recorded every 3 s. Ratiometric images (F_340_/F_380_) were analysed using the Physiology module of AxioVision (Axiovs40 V 4.8.2.0, https://www.zeiss.de/axiovision, Carl-Zeiss, Germany).

### Statistics

All experiments were performed at least three times. Distribution analyses of larvae in temperature preference assays were performed using nonparametric Mann–Whitney U tests to compare two samples and Kruskal–Wallis tests followed by Steel–Dwass tests for multiple comparison. In the analyses of parametric data, Student’s *t*-tests were used to compare two samples, and Dunnett’s tests or Tukey–HSD tests were used to compare three or more samples. Statistical analyses were performed using JMP software or R programmes^78^.

## Supplementary information

Supplementary information
