# Supplementary material for: Functional expression of Δ12 fatty acid desaturase modulates thermoregulatory behaviour in Drosophila
Source: Sci Rep. 2020 Jul 16;10:11798. doi: 10.1038/s41598-020-68601-2 (PMC7366712; doi:10.1038/s41598-020-68601-2)
Supplement: Supplementary file 1 — Supplementary information [file 41598_2020_68601_MOESM1_ESM.pdf]

Supplementary Information

Functional expression of Δ12 fatty acid desaturase modulates thermoregulatory behaviour in *Drosophila*

Takuto Suito[1†], Kohjiro Nagao[1], Kenichi Takeuchi[1], Naoto Juni[1], Yuji Hara[1], Masato Umeda[1*]

[1]Department of Synthetic Chemistry and Biological Chemistry, Graduate School of Engineering, Kyoto University, Katsura, Kyoto 615-8510, Japan

†Present address: Takuto Suito, Division of Thermal Biology, Exploratory Research Center on Life and Living Systems (ExCELLS), National Institutes of Natural Sciences, 5-1 Higashiyama, Myodaiji-cho, Okazaki, Aichi 444-8787, Japan

*To whom correspondence should be addressed: A4-212 Katsura, Nishikyo-ku, Kyoto 615-8510, Japan, Tel.: 81-75-383-2766; Fax.: 81-75-383-2767; E-mail: umeda@sbchem.kyoto-u.ac.jp

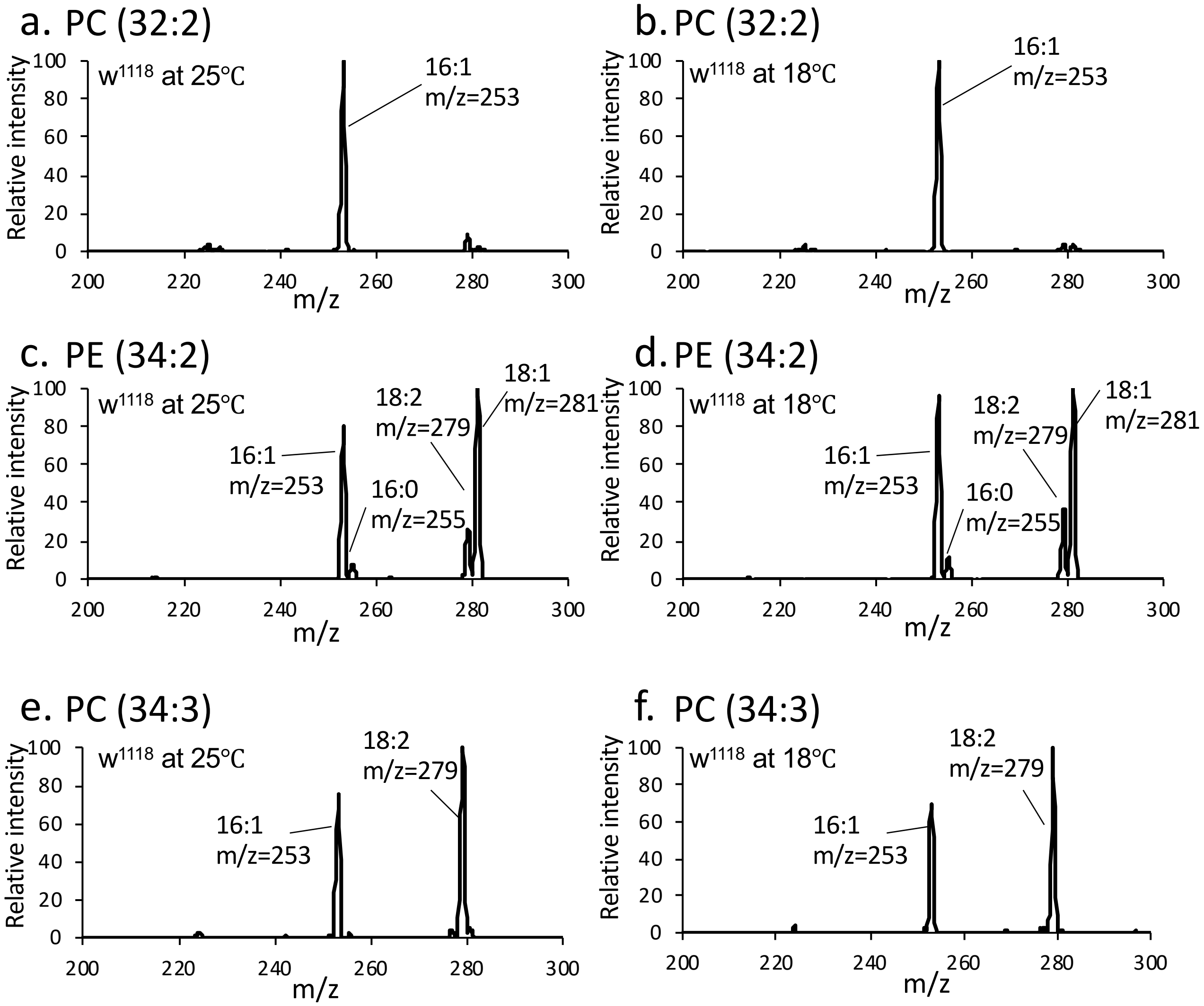

Supplementary Fig. S1. Product ion scan analysis of phospholipids containing more than two double bonds

Product ion scan analyses of PC (32:2) (a, b), PE (34:2) (c, d) and PE (34:3) (e, f) in third instar larvae continuously cultured at 25°C (a, c, e) and exposed to 18°C for 1 day (b, d, f) were performed using LC-MS/MS in negative ion mode. $[M + HCOO]^-$ and $[M - H]^-$ were used as the parent ions of PC and PE, respectively.

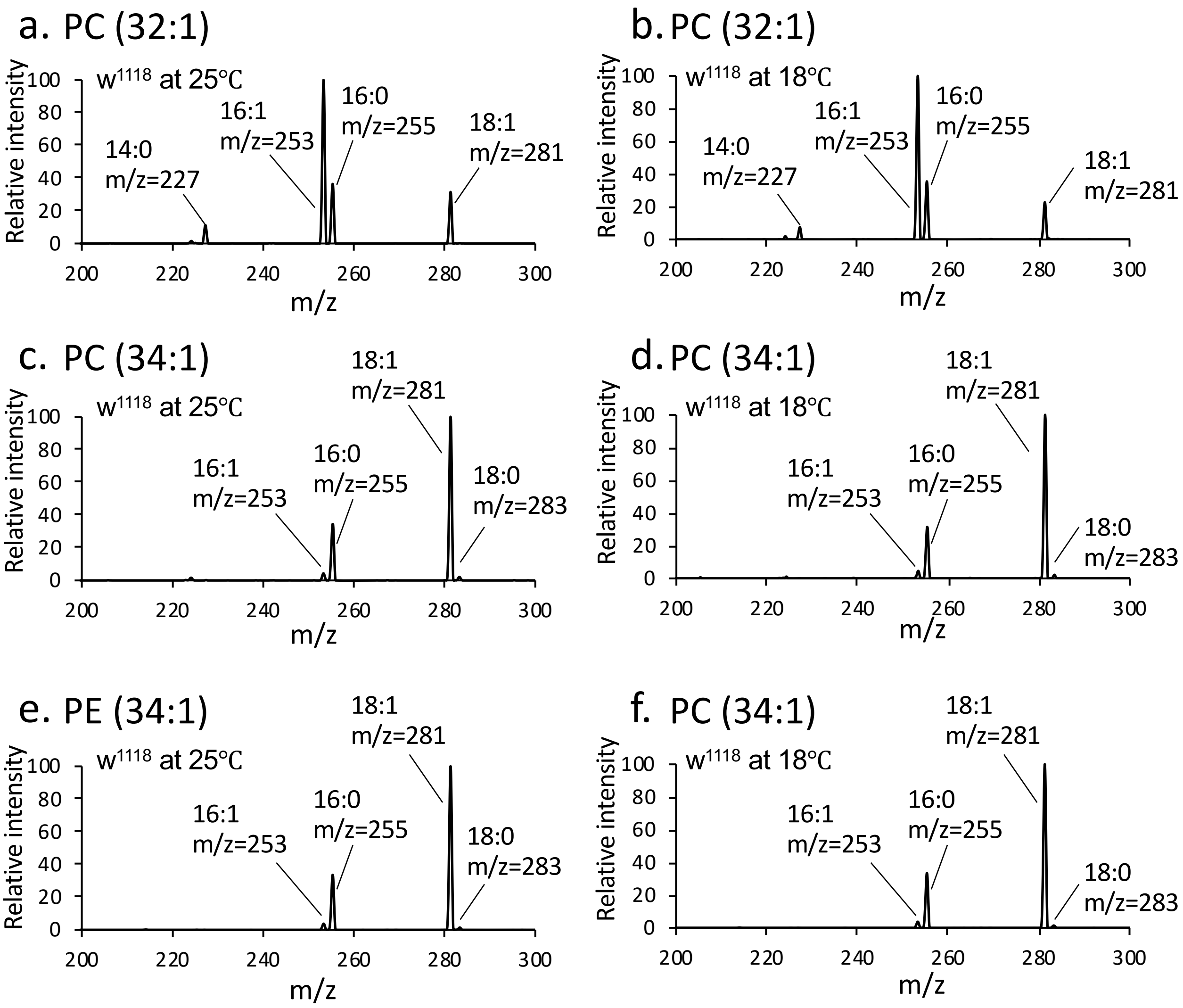

Supplementary Fig. S2. Product ion scan analysis of major phospholipids decreased in cold exposed larvae

Product ion scan analyses of PC (32:1) (a, b), PC (34:1) (c, d) and PE (34:1) (e, f) in third instar larvae continuously cultured at 25°C (a, c, e) and exposed to 18°C for 1 day (b, d, f) were performed using LC-MS/MS in negative ion mode. $[M + HCOO]^-$ and $[M - H]^-$ were used as the parent ions of PC and PE, respectively.

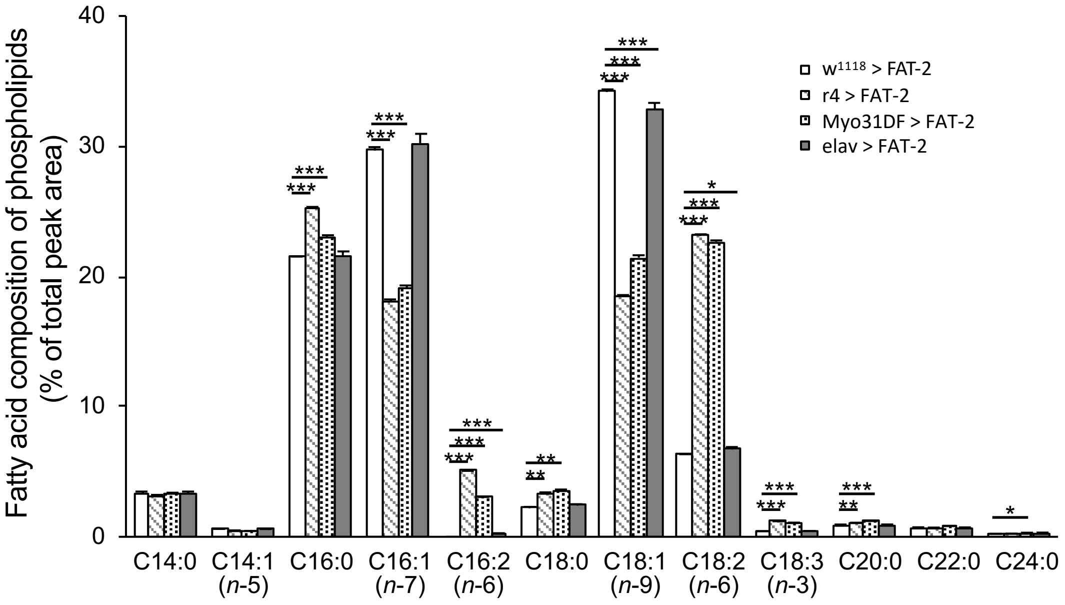

Supplementary Fig. S3. Whole-body phospholipid fatty acid compositions following tissue-specific expression of FAT-2

Whole-body fatty acid compositions were analysed using GC-FID. FAT-2 expression was induced by r4-GAL4 (fat body), Myo31DF-GAL4 (gut), and elav-GAL4 (neuron) (n = 3). Data are presented as means ± SE; * $p < 0.05$; ** $p < 0.01$; *** $p < 0.001$, Dunnett's test.

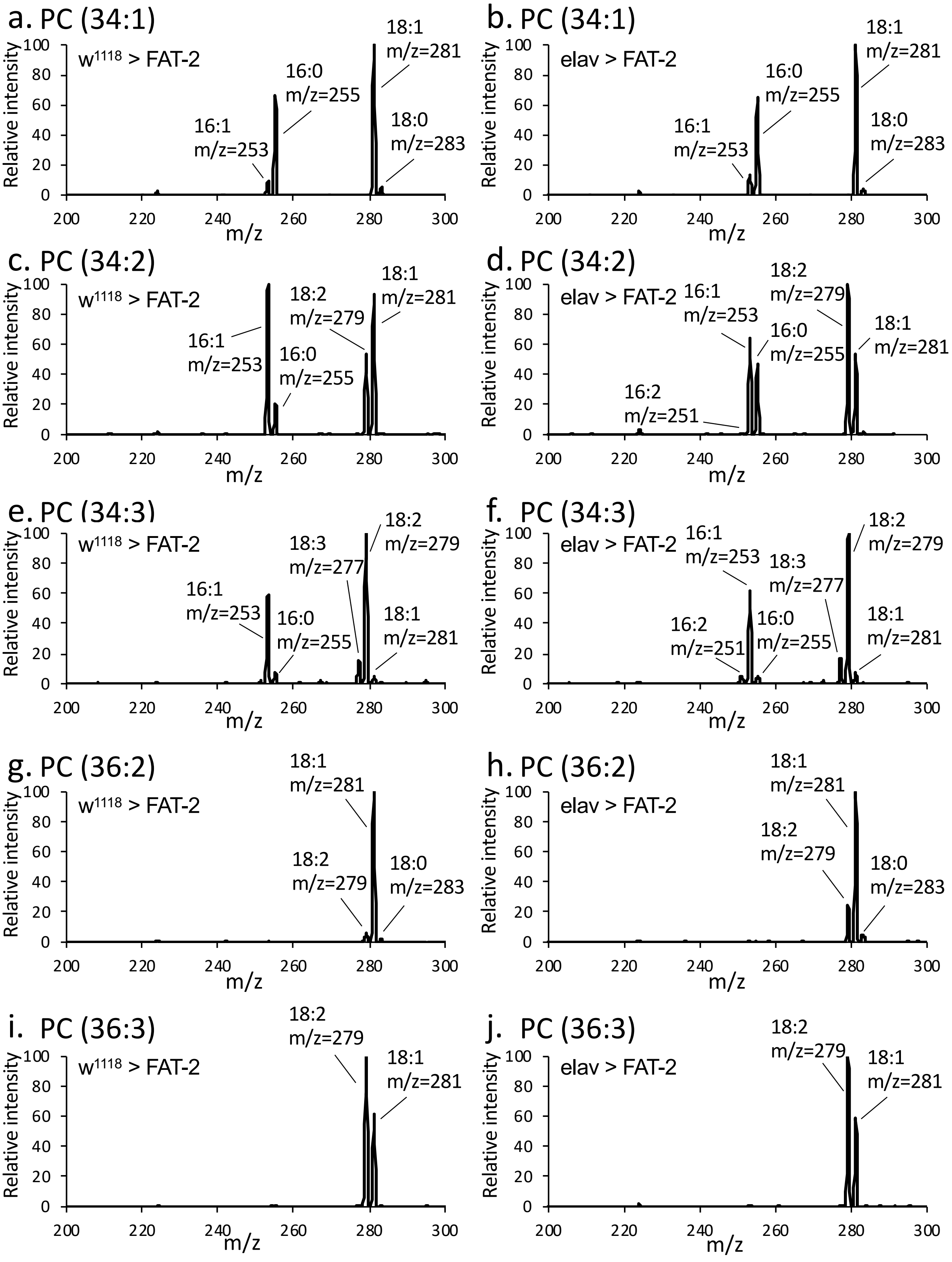

Supplementary Fig. S4. The fatty acid compositions of phosphatidylcholine (PC) in the CNS of neuron-specific FAT-2-expressing larvae

Product ion scan analyses were performed using LC-MS/MS in negative ion mode. MS spectrograms of the $[M + HCOO]^-$ ion of PC (34:1) (a, b), PC (34:2) (c, d), PC (34:3) (e, f), PC (36:2) (g, h) and PC (36:3) (i, j) in w^{1118} > FAT-2 (a, c, e, g, i) or elav > FAT-2 (b, d, f, h, j) flies.

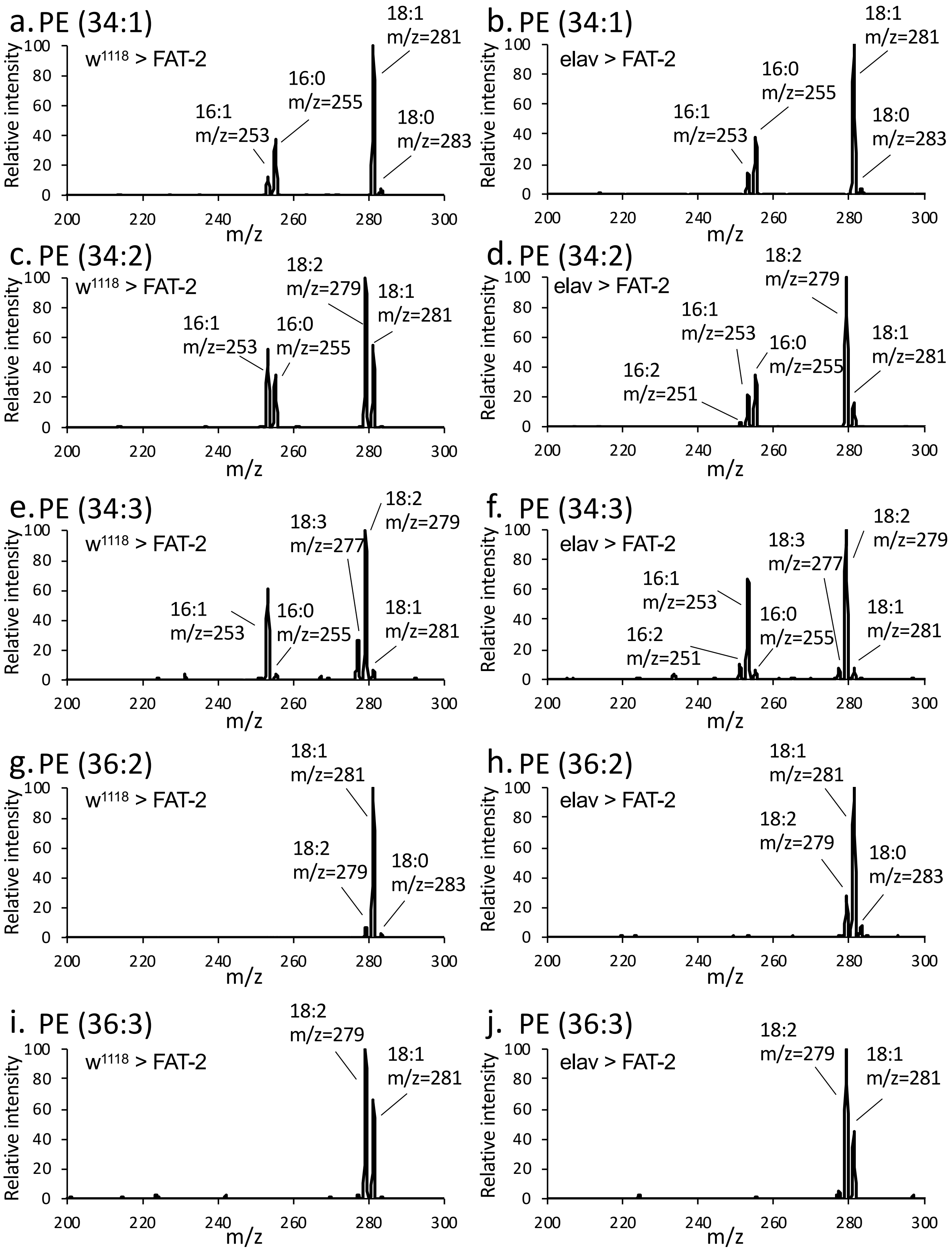

Supplementary Fig. S5. The fatty acid compositions of phosphatidylethanolamine (PE) in the CNS of neuron-specific FAT-2-expressing larvae

Product ion scan analyses were performed using LC-MS/MS in the negative ion mode. MS spectrograms of $[M - H]^-$ ions of PE (34:1) (a, b), PE (34:2) (c, d), PE (34:3) (e, f), PE (36:2) (g, h) and PE (36:3) (i, j) in w^{1118} > FAT-2 (a, c, e, g, i) or elav > FAT-2 (b, d, f, h, j) flies.

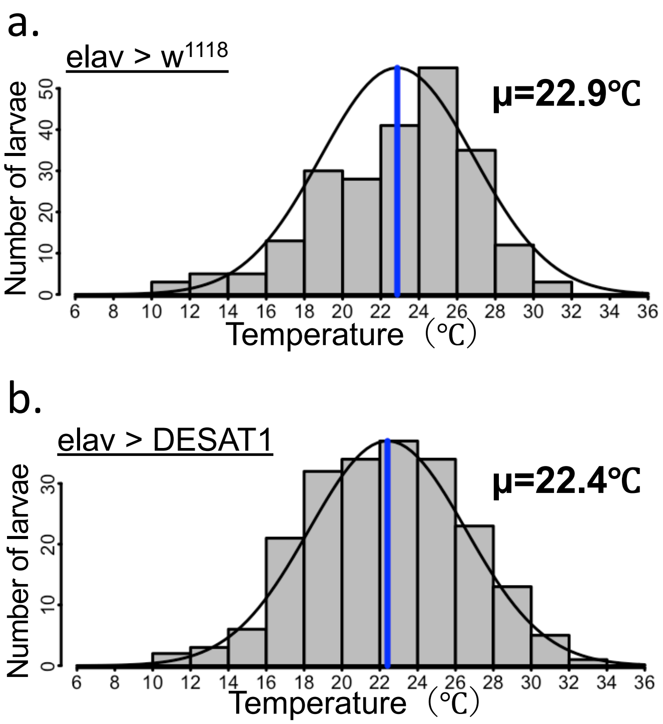

Supplementary Fig. S6. Effects of Δ9 fatty acid desaturase overexpression on temperature preference

The histogram shows distributions of third instar larvae on the thermal gradient plate. The distribution curve is denoted by a black solid line and the average temperature preference is shown as a blue vertical line. The dotted curve represents the distribution of the control (elav > w^{1118}). Temperature preference was measured in third instar larvae expressing Δ9 fatty acid desaturase (DESAT1) under the control of the neuron-specific elav-GAL4 driver (b) (n = 6) and control larvae (a) (n = 6). The numerical analyses of data are also shown in Supplementary Table S3.

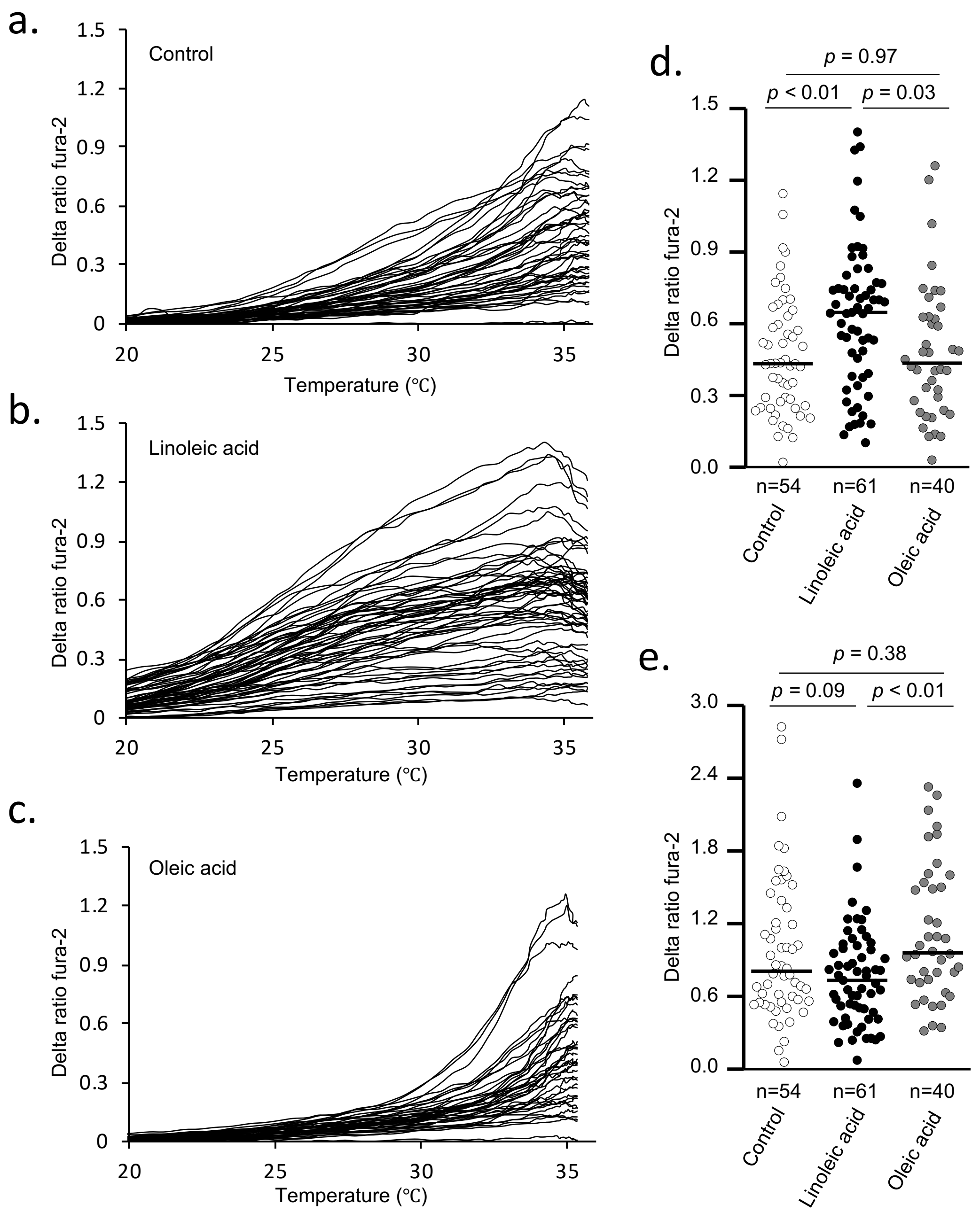

Supplementary Fig. S7. The effects of C18:2 (*n*-6) on temperature response in TRPA1-expressing S2 cells

Temperature responses of TRPA1-expressing *Drosophila* S2 cells were measured according to calcium concentrations. Ratiometric images (F340/F380) were analysed using the Physiology module of AxioVision (Methods). Traces of each cell activity are shown as delta ratios plotted against temperature (a, b, c). Cells were incubated with 100 μM linoleic acid [C18:2 (*n*-6)] (b), 100 μM oleic acid [C18:1 (*n*-9)] (c), or without fatty acids (a) for 6h. The dot plot shows maximum activities induced by warming (d) or 10 mM AITC (e) in control (white dot) cells and cells incubated with C18:2 (*n*-6) (black dot) or C18:1 (*n*-9) (grey dot). The responses against AITC were measured at 20℃ in the same cells that were used for temperature-induced activation. Bars indicate medium values. Statistical analyses were performed using Tukey–HSD tests.

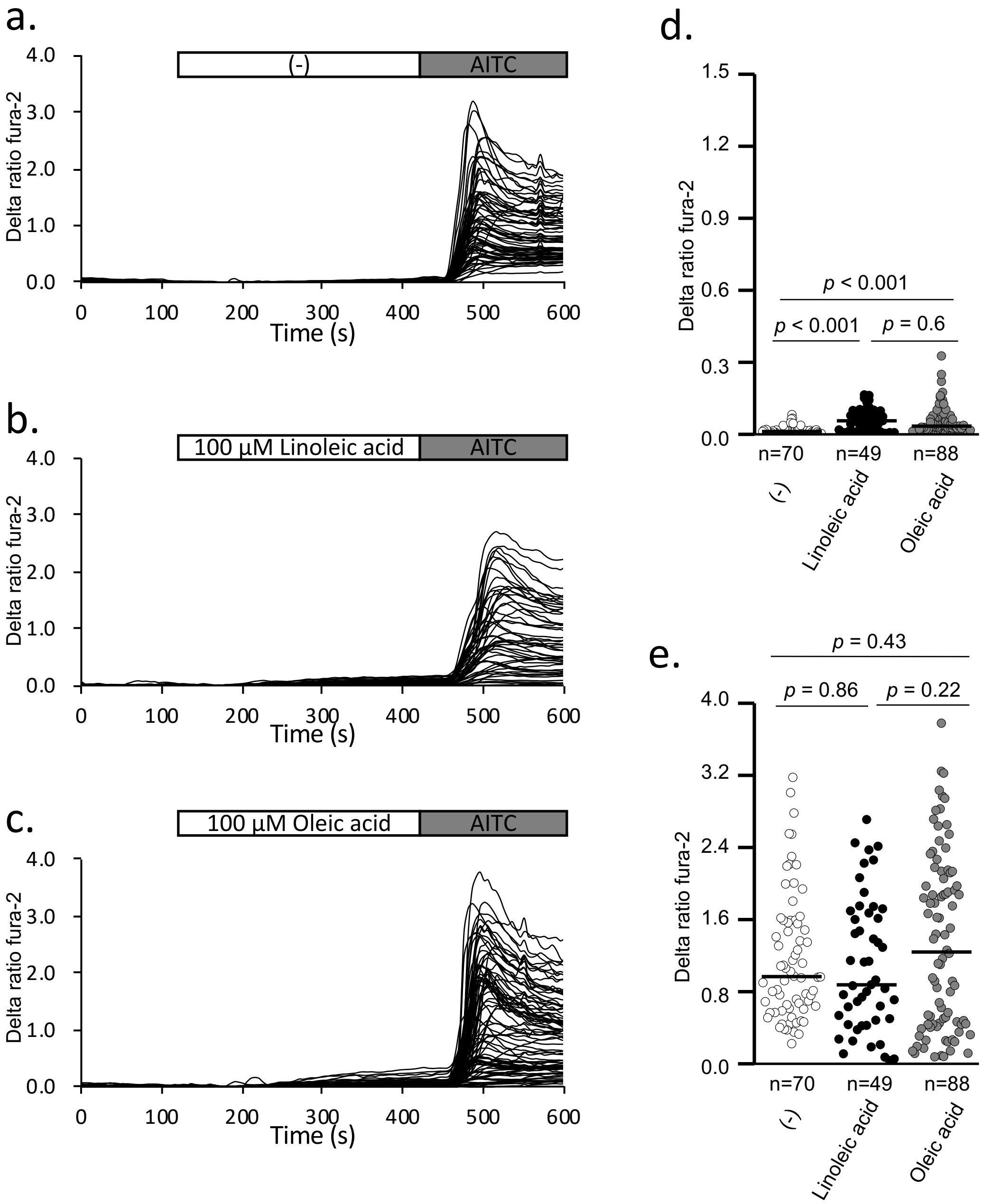

Supplementary Fig. S8. The direct activation of TRPA1-expressing S2 cells by C18:2 (*n*-6)

Responses in control TRPA1-expressing *Drosophila* S2 cells (a), and cells perfused with 100 µM linoleic acid [C18:2 (*n*-6)] (b) and 100 µM oleic acid [C18:1 (*n*-9)] (c) were measured according to changes in calcium concentrations. Ratiometric images (F340/F380) were analysed using the Physiology module of AxioVision (Methods). The dot plot shows maximum activities induced by perfusion of fatty acids (d) and 10 mM AITC (e). The responses against AITC at 20.5°C (control), 20.4°C (C18:2 (*n*-6)), and 21.5°C (C18:1 (*n*-9)) were measured in the same cells after perfusion of fatty acids. White, black, and grey dots represent cells that were perfused with nothing (control), C18:2 (*n*-6) and C18:1 (*n*-9), respectively. Bars indicate median values. Statistical analyses were performed using Tukey–HSD tests.

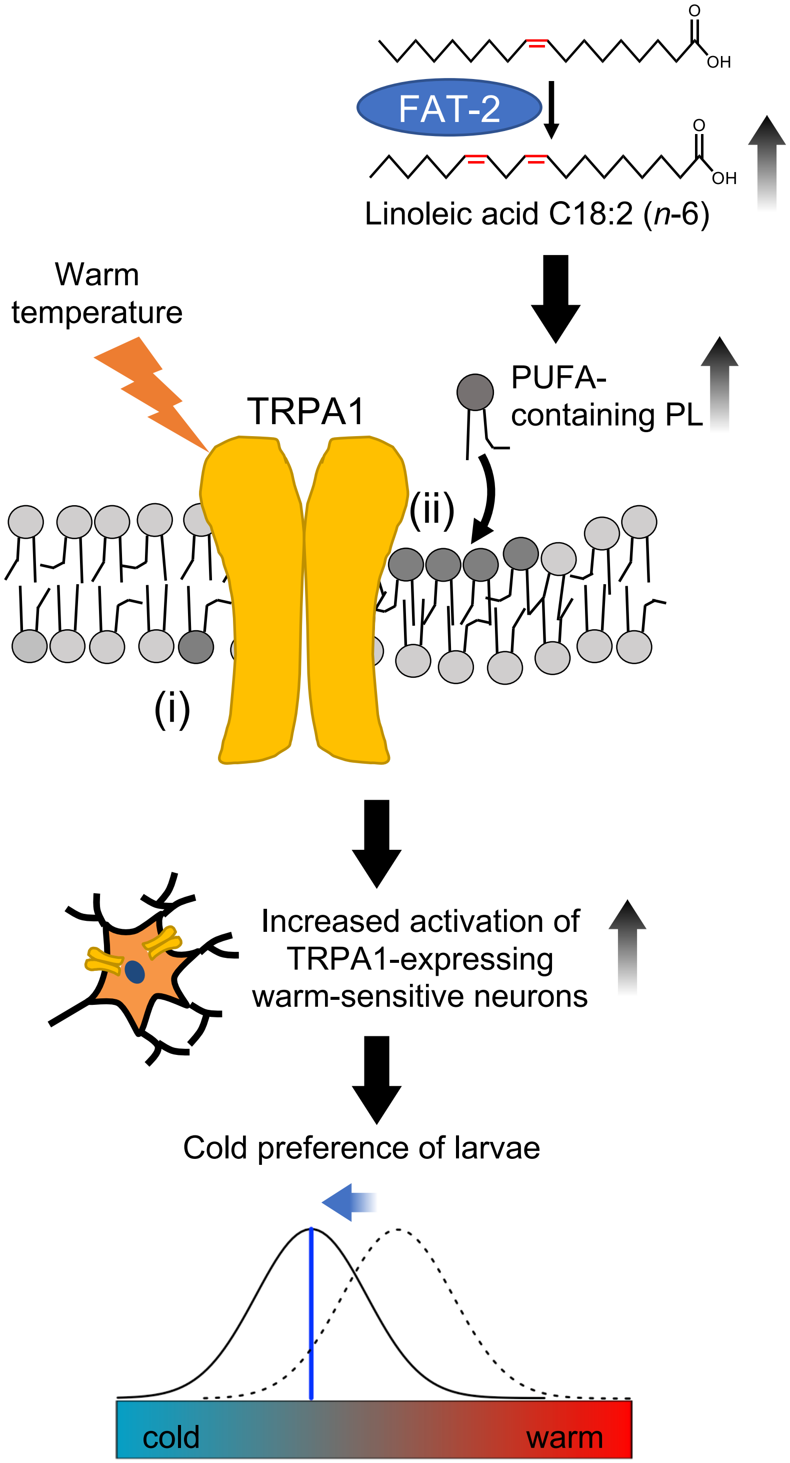

Supplementary Fig. S9. The putative models for FAT-2-induced regulation of thermoregulatory behavior

FAT-2 expression in TRPA1-expressing warm-sensitive neuron leads to the biosynthesis of C18:2 (*n*-6) and increases the proportion of C18:2 (*n*-6)-containing phospholipids in the lipid membrane. The C18:2 (*n*-6)-containing phospholipids enhance the activity of thermoreceptor channel TRPA1 via (i) direct interaction with TRPA1 or (ii) modulating the physicochemical properties of the lipid membrane. The increased activation of TRPA1-expressing warm-sensitive neurons causes cold preference in larvae.

Supplementary Table S1. Temperature preference of the w^{1118} third instar larvae incubated at 25°C and 18°C

Flies	Preferred temperature μ (°C), σ^2		Statistical Significance
w^{1118} at 25°C	$\mu = 22.5$	$\sigma^2 = 3.3^2$	
w^{1118} at 18°C	$\mu = 20.7$	$\sigma^2 = 4.1^2$	***

*** $p < 0.001$, Mann–Whitney U test.

Supplementary Table S2. Temperature preference of third instar larvae expressing FAT-2 under the control of various tissue-specific GAL4 drivers

Flies	Preferred temperature μ (°C), σ^2		Statistical Significance
w^{1118}	$\mu = 21.8$	$\sigma^2 = 3.7^2$	
w^{1118} > FAT-2	$\mu = 20.8$	$\sigma^2 = 4.2^2$	
tub > FAT-2	$\mu = 21.9$	$\sigma^2 = 5.0^2$	
tub > w^{1118}	$\mu = 23.3$	$\sigma^2 = 3.8^2$	
r4 > FAT-2	$\mu = 19.6$	$\sigma^2 = 4.4^2$	†, #
r4 > w^{1118}	$\mu = 21.5$	$\sigma^2 = 4.3^2$	
Myo31DF > FAT-2	$\mu = 20.4$	$\sigma^2 = 4.8^2$	#
Myo31DF > w^{1118}	$\mu = 22.3$	$\sigma^2 = 4.1^2$	
elav > FAT-2	$\mu = 18.6$	$\sigma^2 = 4.5^2$	†, #, *
elav > w^{1118}	$\mu = 22.1$	$\sigma^2 = 4.0^2$	

The FAT-2-expressing flies with †, # or * indicate that their preferred temperature is significantly different from that of w^{1118} control flies, tissue-specific GAL-4 crossed with w^{1118}, or UAS-FAT-2 crossed with w^{1118}, respectively. (Steel–Dwass test, $p < 0.05$).

Supplementary Table S3. Effects of Δ9 fatty acid desaturase overexpression on temperature preference

Flies	Preferred temperature μ (°C), σ^2	
elav > w^{1118}	$\mu = 22.9$	$\sigma^2 = 4.0^2$
elav > DESAT1	$\mu = 22.4$	$\sigma^2 = 4.1^2$

No significant difference was observed using Mann–Whitney U test.

Supplementary Table S4. Temperature preference of third instar larvae expressing FAT-2 under the control of various thermoreceptor-expressing cell-specific GAL4 drivers

Flies	Preferred temperature μ (°C), σ^2		Statistical Significance
w^{1118} > FAT-2	$\mu = 21.7$	$\sigma^2 = 4.0^2$	
TRPA1 > FAT-2	$\mu = 17.3$	$\sigma^2 = 5.0^2$	#, *
TRPA1 > w^{1118}	$\mu = 22.2$	$\sigma^2 = 3.1^2$	
iav > FAT-2	$\mu = 19.3$	$\sigma^2 = 3.7^2$	#, *
iav > w^{1118}	$\mu = 22.1$	$\sigma^2 = 3.7^2$	
TRP > FAT-2	$\mu = 19.1$	$\sigma^2 = 4.8^2$	#, *
TRP > w^{1118}	$\mu = 22.9$	$\sigma^2 = 4.1^2$	
TRPL > FAT-2	$\mu = 19.7$	$\sigma^2 = 4.9^2$	*
TRPL > w^{1118}	$\mu = 21.5$	$\sigma^2 = 3.5^2$	
R11F02 > FAT-2	$\mu = 18.8$	$\sigma^2 = 3.6^2$	#, *
R11F02 > w^{1118}	$\mu = 21.2$	$\sigma^2 = 4.1^2$	

The FAT-2-expressing flies with # or * indicate that their preferred temperature is significantly different from that of thermoreceptor-expressing cell-specific GAL-4 crossed with w^{1118} or UAS-FAT-2 crossed with w^{1118}, respectively. (Steel–Dwass test, $p < 0.05$).

Supplementary Table S5. Temperature preference of third instar larvae expressing FAT-2 in distinct TRPA1-expressing neurons

Flies	Preferred temperature μ (°C), σ^2		Statistical Significance
w^{1118} > FAT-2	$\mu = 21.2$	$\sigma^2 = 4.2^2$	
TRPA1-AB > FAT-2	$\mu = 18.8$	$\sigma^2 = 4.7^2$	#, *
TRPA1-AB > w^{1118}	$\mu = 23.8$	$\sigma^2 = 4.9^2$	
TRPA1-CD > FAT-2	$\mu = 19.0$	$\sigma^2 = 4.2^2$	#, *
TRPA1-CD > w^{1118}	$\mu = 22.1$	$\sigma^2 = 3.5^2$	

The FAT-2-expressing flies with # or * indicate that their preferred temperature is significantly different from that of TRPA1-expressing neuron-specific GAL-4 crossed with w^{1118} or UAS-FAT-2 crossed with w^{1118}, respectively. (Steel–Dwass test, $p < 0.05$).

Supplementary Table S6. Temperature preference of third instar larvae expressing FAT-2 and tetanus toxin under the control of TRPA1-expressing neuron-specific driver expressing

Flies	Preferred temperature μ (°C), σ^2		Statistical Significance
TRPA1 > w^{1118}	$\mu = 22.1$	$\sigma^2 = 3.7^2$	
TRPA1 > FAT-2	$\mu = 17.8$	$\sigma^2 = 4.3^2$	#
TRPA1 > TeTxLC	$\mu = 27.7$	$\sigma^2 = 3.0^2$	#
TRPA1 > TeTxLC; FAT-2	$\mu = 27.9$	$\sigma^2 = 2.7^2$	#

The flies with # indicate that their preferred temperature is significantly different from that of TRPA1-GAL-4 crossed with w^{1118}. (Steel–Dwass test, $p < 0.05$). No significant difference was observed between TRPA1 > TeTxLC and TRPA1 > TeTxLC; FAT-2 using Steel–Dwass test.
